# Exact Analytical Solutions for Static Response of Helical Single-Walled Carbon Nanotubes Using Nonlocal Euler–Bernoulli Beam Theory

**DOI:** 10.3390/nano15191461

**Published:** 2025-09-23

**Authors:** Ali Murtaza Dalgıç, Mertol Tüfekci, İnci Pir, Ekrem Tüfekci

**Affiliations:** 1 Faculty of Mechanical Engineering, Istanbul Technical University, Istanbul 34437, Türkiye; dalgica17@itu.edu.tr (A.M.D.); pirin@itu.edu.tr (İ.P.); tufekcie@itu.edu.tr (E.T.); 2 Centre for Engineering Research, University of Hertfordshire, Hatfield AL109AB, UK; 3 School of Physics, Engineering and Computer Science, University of Hertfordshire, Hatfield AL109AB, UK

**Keywords:** helical single-walled carbon nanotube beams, nanomechanics, nonlocal elasticity theory, static behaviour, exact analytical solution, initial value

## Abstract

This study presents an exact analytical investigation into the static response of helical single-walled carbon nanotube (SWCNT) beams based on Eringen’s differential nonlocal elasticity theory, which captures nanoscale effects arising from interatomic interactions. A key contribution of this work is the derivation of the governing equations for helical SWCNT beams, based on the nonlocal Euler–Bernoulli theory, followed by their exact analytical solution using the initial value method. To the best of the authors’ knowledge, this represents the first closed-form formulation for such complex nanostructures using this theoretical framework of nonlocal elasticity theory. The analysis considers both cantilevered and clamped–clamped boundary conditions, under various concentrated force and moment loadings applied at the ends and midpoint of the helical beam. Displacements and rotational components are expressed in the Frenet frame, enabling direction-specific evaluation of the deformation behaviour. Parametric studies are conducted to investigate the influence of geometric parameters—such as the winding angle (α) and aspect ratio (R/d) and the nonlocal parameter (R/γ). Results show that nonlocal elasticity theory consistently predicts higher displacements and rotations than the classical local theory, revealing its importance for accurate modelling of nanoscale structures. The proposed analytical framework serves as a benchmark reference for the modelling and design of nanoscale helical structures such as nano-springs, actuators, and flexible nanodevices.

## 1. Introduction

The principles of classical continuum mechanics are inadequate in capturing size-dependent effects in nanoscale structures such as nanobeams and nanorods. Consequently, various theoretical frameworks have been proposed to address this insufficiency, including molecular dynamics, statistical mechanics, doublet mechanics, and nonlocal elasticity theories. Among these are Eringen’s nonlocal elasticity theory, strain-driven and stress-driven nonlocal models, modified strain gradient theories, and surface elasticity theories [1,2,3,4,5,6,7,8,9].

The relationship between statistical mechanics and nonlocal mechanics theories shows the significance and need for a general theory of particle dynamics, which would combine concepts and improve the understanding of nonlocal interactions [10,11]. The discussion regarding the mechanical response of nanobeams remains ongoing, as research indicates that nonlocal effects can result in either stiffening or softening behaviour, depending on the specific loading conditions and structural configurations [12,13]. Scale effects on structural behaviour are demonstrated through exact solutions for bending deflections in nonlocal elasticity theory [14]. Studies of nonlocal scalar field theories inspired by string theory have opened new perspectives on material behaviour [15].

Molecular dynamics simulations are evaluated in nano-scale structure problems. With bridging gaps between simulations and continuum models, calibrating nonlocal generalised helical beam models for free vibration analysis of coiled carbon nanotubes is one of the examples in the literature [16].

Advanced nonlocal elasticity theories have also been developed to solve nanostructure problems. A review of nonlocal elasticity theories reveals the significance of these theories in material science, highlighting the ongoing advancements and developments [17]. The nonlocal strain gradient theory based on the Bishop rod model is one of the examples of applications that capture complex behaviours in nanostructures [18]. The strain-driven nonlocal model has been employed to examine the static behaviour of nanobeams with edge cracks. This approach has yielded insights into the impact of cracks on the mechanical properties of nanoscale structures [19]. The model has also been applied to bending and buckling analyses of curved nanobeams. These analyses utilise higher-order beam theories, demonstrating the influence of nonlocal effects on stability and deformation [20,21,22].

Advanced solution methods, such as the Laplace-Differential Transformation Method, have been employed alongside nonlocal elasticity theory to investigate the large bending behaviour of carbon nanotubes, providing precise solutions while significantly improving computational efficiency [23]. To emphasise the importance of size effects in design and analysis, the strain-driven nonlocal elasticity theory has been utilised in several nanostructure problems [24]. With the modifications to the nonlocal strain gradient model, the size-dependent bending behaviour of Timoshenko curved beams has been developed [25]. Eringen’s nonlocal elasticity theory is essential in analysing nanoscale structures. The methodology has been implemented in the context of dynamic analyses of curved nanobeams, employing higher-order beam theories. This approach has been shown to facilitate accounting for small-scale effects and complex geometries, thereby ensuring a comprehensive and nuanced understanding of the subject matter [20].

Furthermore, Polizzotto et al. [26] examined absolute and relative size effects within nonlocal strain-gradient elastic beams, while Giorgio [27] introduced a discrete formulation of Kirchhoff rods in large-motion dynamics. These studies highlight the diversity of available approaches, yet none provide an exact closed-form solution for helical nanotubes. The present work therefore fills this gap by developing a rigorous analytical framework in Frenet coordinates, establishing a benchmark for future investigations.

A two-phase local/nonlocal elasticity theory has been developed for the purpose of analysing the free transverse vibration of rotating nanobeams. This comprehensive modelling approach can be used to study rotating nanostructures [28]. Nonlocal Timoshenko beam theory has been employed to investigate layered and heterogeneous microstructures, thereby enhancing the modelling of composite nanostructures [29].

An investigation has been conducted into the vibrational behaviour of piezoelectric nanobeams, taking into account the impact of surface effects and nonlocal elasticity [3,30]. A thorough analysis of clamped nanobeams as adsorption-induced sensors has been conducted, addressing both linear and nonlinear effects due to surface stresses generated by particle adsorption and desorption [31].

The applications of this field extend to biological structures. Research on the mechanical behaviour of protein microtubules using nonlocal elasticity theory has enriched the cellular biomechanics field [32]. Highlighting the impact of curvature and nonlocal effects, curved carbon nanotubes’ vibration and stability behaviours have been studied [33]. For the purpose of conducting static and dynamic analysis of nanobeams under axial load, A generalised formulation of Eringen’s nonlocal elasticity theory has been developed [34,35].

The use of two-phase nonlocal integral models to conduct free vibration analysis of curved Euler–Bernoulli beams has been demonstrated to enhance the accuracy of predictions of dynamic behaviour [36]. Calibrating nonlocal elasticity models with experimental data has a significant place. The stress-driven nonlocal elasticity model has been validated using experimental data on the bending and free vibration of micro- and nanocantilevers [2]. The free vibrations of stepped nanobeams with cracks have been studied using Eringen’s nonlocal elasticity theory [37].

Curved beams are essential for designing compliant mechanisms. A six-degree-of-freedom compliant mechanism based on curved beams has been proposed. It demonstrates multi-degrees-of-freedom motion with simple structures using isogeometric analysis [38]. To address the problem of large deflections in beams, a comprehensive static modelling methodology has been developed using beam theory for compliant mechanisms [39]. Using variational principles and differential geometry, the vibration of thin pre-twisted helical beams has been studied, and the governing equations have been established [40]. The static behaviour of nanobeams under variable loads has been examined using analytical solutions and nonlocal elasticity theory in Frenet coordinates, addressing the constraints of conventional local elasticity theories in nanoscale systems [41,42]. An analytical approach examines the bending behaviour of curved double-walled nanotubes [9]. Similar methodologies are followed in the buckling prediction in single-walled nanotubes [43] and double-walled nanotubes [44].

The literature on helical carbon nanotubes is currently limited. However, they have attracted considerable attention due to their unique structural and mechanical properties [45]. In order to regulate their formation and improve their performance, several synthesis techniques have been developed [46]. Their mechanical properties, such as tensile strength and crack resistance, have been investigated through both experimental and modelling techniques [47]. In addition, these nanotubes are being incorporated into energy storage devices and composite materials to enhance functionality [48,49].

This study formulates Eringen’s nonlocal elasticity theory within the framework of Euler–Bernoulli beam theory expressed in Frenet coordinates to analyse the static behaviour of helical single-walled carbon nanotubes (SWCNTs) modelled as nanobeams [50,51]. The nonlocal framework introduces size-dependent effects absent in classical continuum models, enabling the governing equations to capture interatomic interactions. The resulting system of differential equations is solved exactly using the initial value method, yielding closed-form expressions for displacements and rotations under various loading conditions. By comparing local and nonlocal predictions, the analysis provides deeper insight into the influence of nanoscale interactions on the deformation of helical nanostructures. The novelty of this work lies in presenting the first exact closed-form formulation for the nonlocal static response of helical SWCNT beams, with the examples used to demonstrate the analytical solutions presented as parametric studies covering different geometries and length scales, thereby providing benchmark results for nanoscale device design.

## 2. The Governing Differential Equations for Static Behaviour of Helical SWCNTs

The helical SWCNT is described using beam theory together with expressions obtained from nonlocal elasticity. Its centreline is assumed to trace a helical curve characterised by a constant radius and a fixed pitch angle, denoted by α. Hence the Frenet coordinate system is employed. In the Frenet frame, the tangent (t), normal (n), and binormal (b) directions provide a natural basis aligned with the helix geometry. Resolving displacements and rotations along these directions allows the formulation to capture the intrinsic coupling between axial elongation, bending, and torsion in a compact mathematical form. This geometric description helps connect the forthcoming algebraic derivations to physical deformation modes of the helix. Furthermore, the cross-section is considered uniform and doubly symmetric, without any initial twist. The corresponding parametric form of the helix is well established in the literature and can be expressed as:

(1) x=Rcos⁡α

(2) y=Rsin⁡α

(3) z=Rθtan⁡α

(4) c=R/cos⁡α

Here, θ denotes the helix angle, α represents the pitch angle, and R is the helix radius. The parameter c is a scaling factor that links the arc length to the helix angle, facilitating the geometric description of the helix.

**Figure 1:**
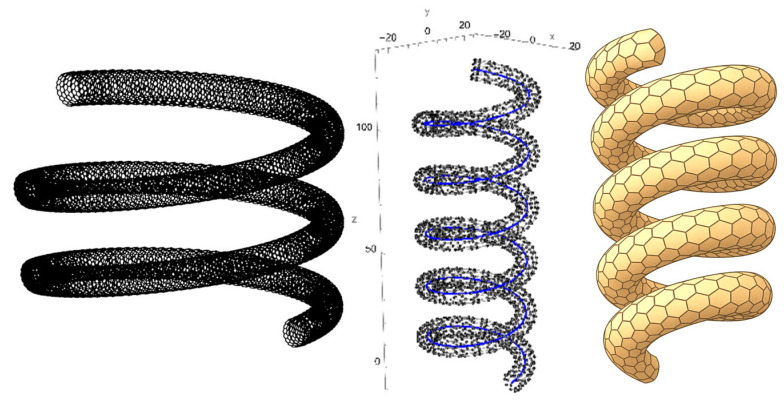
Representative models of a helical single-walled carbon nanotube (SWCNT): atomistic configuration (**left**, **middle**), and continuum model (**right**).

A schematic of a helical SWCNT with a uniform circular cross-section, constant radius, and fixed pitch angle is illustrated in Figure 1.

According to Eringen’s formulation, the relationship between stresses in the classical (local) and nonlocal elasticity theories is expressed as:

(5) $$\left(1-\gamma^{2}\nabla^{2}\right)\sigma^{nl}=\sigma^{l}$$

where $\nabla^{2}$ denotes the Laplacian operator, γ stands for the nonlocal parameter, $\sigma^{l}$ and $\sigma^{nl}$ indicate the stress tensors in local (classical) and nonlocal elasticity theories, respectively.

The correspondence between local and nonlocal force and moment resultants, as reported in the literature [41,42,52,53,54], can be written as:

(6) $$F_{n}^{nl}-\frac{\gamma^{2}}{R^{2}}\frac{d^{2}F_{n}^{nl}}{d\theta^{2}}=F_{n}^{l}$$

(7) $$(1+\frac{\gamma^{2}}{R^{2}})F_{b}^{nl}-\frac{\gamma^{2}}{R^{2}}\frac{d^{2}F_{b}^{nl}}{d\theta^{2}}=F_{b}^{l}$$

(8) $$F_{t}^{nl}-\frac{\gamma^{2}}{R^{2}}\frac{d^{2}F_{t}^{nl}}{d\theta^{2}}=F_{t}^{l}$$

(9) $$M_{n}^{nl}-\frac{\gamma^{2}}{R^{2}}\frac{d^{2}M_{n}^{nl}}{d\theta^{2}}=M_{n}^{l}$$

(10) $$M_{b}^{nl}-\frac{\gamma^{2}}{R^{2}}\frac{d^{2}M_{b}^{nl}}{d\theta^{2}}+\frac{\gamma^{2}}{R}F_{t}^{nl}=M_{b}^{l}$$

(11) $$(1+\frac{\gamma^{2}}{R^{2}})M_{t}^{nl}-\frac{\gamma^{2}}{R^{2}}\frac{d^{2}M_{t}^{nl}}{d\theta^{2}}+\frac{\gamma^{2}}{2R^{2}}\frac{dM_{n}^{nl}}{d\theta }+\frac{\gamma^{2}}{2R}F_{b}^{nl}=M_{t}^{l}$$

where $F_{n}^{nl}$, $F_{b}^{nl}$, and $F_{t}^{nl}$ indicate the nonlocal force resultants, $F_{n}^{l}$, $F_{b}^{l}$ and $F_{t}^{l}$ denote the local force resultants, $M_{n}^{nl}$, $M_{b}^{nl}$ and $M_{t}^{nl}$ stand for the nonlocal moment resultants, $M_{n}^{l}$, $M_{b}^{l}$ and $M_{t}^{l}$ are the local moment resultants. The subscripts n, b, and t correspond to the normal, binormal, and tangential directions of the Frenet frame along the helix axis, respectively.

The equilibrium equations for beams, as established in the literature [55,56], are expressed as:

(12) $$\frac{dM_{n}^{nl}}{d\theta }=\sin\alpha M_{b}^{nl}-\cos\alpha M_{t}^{nl}+\frac{R}{\cos\alpha }F_{b}^{nl}$$

(13) $$\frac{dM_{b}^{nl}}{d\theta }=-\sin\alpha M_{n}^{nl}-\frac{R}{\cos\alpha }F_{n}^{nl}$$

(14) $$\frac{dM_{t}^{nl}}{d\theta }=\cos\alpha M_{n}^{nl}$$

(15) $$\frac{dF_{n}^{nl}}{d\theta }=\sin\alpha F_{b}^{nl}-\cos\alpha F_{t}^{nl}$$

(16) $$\frac{dF_{b}^{nl}}{d\theta }=-\sin\alpha F_{n}^{nl}$$

(17) $$\frac{dF_{t}^{nl}}{d\theta }=\cos\alpha F_{n}^{nl}$$

Derivation of Equations (12)–(17) yields:

(18) $$\frac{d^{2}M_{n}^{nl}}{{d\theta }^{2}}=\sin\alpha \frac{dM_{b}^{nl}}{d\theta }-\cos\alpha \frac{dM_{t}^{nl}}{d\theta }+\frac{R}{\cos\alpha }\frac{dF_{b}^{nl}}{d\theta }$$

(19) $$\frac{d^{2}M_{b}^{nl}}{{d\theta }^{2}}=-\sin\alpha \frac{dM_{n}^{nl}}{d\theta }-\frac{R}{\cos\alpha }\frac{dF_{n}^{nl}}{d\theta }$$

(20) $$\frac{d^{2}M_{t}^{nl}}{d\theta^{2}}=\cos\alpha \frac{dM_{n}^{nl}}{d\theta }$$

(21) $$\frac{d^{2}F_{n}^{nl}}{{d\theta }^{2}}=\sin\alpha \frac{dF_{b}^{nl}}{d\theta }-\cos\alpha \frac{dF_{t}^{nl}}{d\theta }$$

(22) $$\frac{d^{2}F_{b}^{nl}}{{d\theta }^{2}}=-\sin\alpha \frac{dF_{n}^{nl}}{d\theta }$$

(23) $$\frac{d^{2}F_{t}^{nl}}{{d\theta }^{2}}=\cos\alpha \frac{dF_{n}^{nl}}{d\theta }$$

Inserting Equations (12)–(23) into Equations (6)–(11) leads to the expressions below, which hold for helices of constant radius, uniform cross-section, and fixed pitch angle.

(24) $$\left(1+\frac{\gamma^{2}}{R^{2}}\right)F_{n}^{nl}=F_{n}^{l}$$

(25) $$\left[1+\frac{\gamma^{2}}{R^{2}}\left[1+{\left(\sin\alpha \right)}^{2}\right]\right]F_{b}^{nl}-\frac{\gamma^{2}}{R^{2}}\sin\alpha \cos\alpha F_{t}^{nl}=F_{b}^{l}$$

(26) $$\left[1+\frac{\gamma^{2}}{R^{2}}{\left(\cos\alpha \right)}^{2}\right]F_{t}^{nl}-\frac{\gamma^{2}}{R^{2}}\cos\alpha \sin\alpha F_{b}^{nl}=F_{t}^{l}$$

(27) $$\left(1+\frac{\gamma^{2}}{R^{2}}\right)M_{n}^{nl}+\frac{\gamma^{2}}{R^{2}}\frac{2R\sin\alpha }{\cos\alpha }F_{n}^{nl}=M_{n}^{l}$$

(28) $$\left(1+\frac{\gamma^{2}}{R^{2}}{\left(\sin\alpha \right)}^{2}\right)M_{b}^{nl}-\frac{\gamma^{2}}{R^{2}}\sin\alpha \cos\alpha M_{t}^{nl}+2\frac{\gamma^{2}}{R}\frac{\sin\alpha }{\cos\alpha }F_{b}^{nl}=M_{b}^{l}$$

(29) $$\begin{array}{c} \left(1+\frac{\gamma^{2}}{R^{2}}\left(1+{\left(\cos\alpha \right)}^{2}-\frac{\cos\alpha }{2}\right)\right)M_{t}^{nl}+\frac{\gamma^{2}}{R^{2}}\sin\alpha \left(\frac{1}{2}-\cos\alpha \right)M_{b}^{nl}\\ +\frac{\gamma^{2}}{2R}\left(\frac{1}{\cos\alpha }-1\right)F_{b}^{nl}=M_{t}^{l} \end{array}$$

From these relations, the governing differential equations for the static response of helical SWCNTs subjected to concentrated forces are derived [57]:

(30) $$\frac{du_{n}^{nl}\left(\theta \right)}{d\theta }=\sin\alpha u_{b}^{nl}-\cos\alpha u_{t}^{nl}+\frac{R}{\cos\alpha }\Omega_{b}^{nl}$$

(31) $$\frac{du_{b}^{nl}\left(\theta \right)}{d\theta }=-\sin\alpha u_{n}^{nl}-\frac{R}{\cos\alpha }\Omega_{n}^{nl}$$

(32) $$\frac{du_{t}^{nl}\left(\theta \right)}{d\theta }=\cos\alpha u_{n}^{nl}$$

(33) $$\frac{d\Omega_{n}^{nl}\left(\theta \right)}{d\theta }=\sin\alpha \Omega_{b}^{nl}-\cos\alpha \Omega_{t}^{nl}+\left(1+\frac{\gamma^{2}}{R^{2}}\right)\frac{R}{\cos\alpha EI_{n}}M_{n}^{nl}+\frac{\gamma^{2}}{R^{2}}\frac{2R^{2}\sin\alpha }{EI_{n}{\left(\cos\alpha \right)}^{2}}F_{n}^{nl}$$

(34) $$\begin{array}{c} \frac{d\Omega_{b}^{nl}\left(\theta \right)}{d\theta }=-\sin\alpha \Omega_{n}^{nl}\\ \;\;+\frac{R}{\cos\alpha EI_{b}}[\left(1+\frac{\gamma^{2}}{R^{2}}{\left(\sin\alpha \right)}^{2}\right)M_{b}^{nl}-\frac{\gamma^{2}}{R^{2}}\sin\alpha \cos\alpha M_{t}^{nl}\\ \;\;+2\frac{\gamma^{2}}{R^{2}}\frac{\sin\alpha }{\cos\alpha }RF_{b}^{nl}] \end{array}$$

(35) $$\begin{array}{c} \frac{d\Omega_{t}^{nl}\left(\theta \right)}{d\theta }=\cos\alpha \Omega_{n}^{nl}+\frac{R}{\cos\alpha GJ}\left[1+\frac{\gamma^{2}}{R^{2}}\left(1+{\left(\cos\alpha \right)}^{2}-\frac{\cos\alpha }{2}\right)\right]M_{t}^{nl}\\ +\frac{R}{\cos\alpha GJ}\frac{\gamma^{2}}{R^{2}}\left[\sin\alpha \left(\frac{1}{2}-\cos\alpha \right)M_{b}^{nl}+\frac{R}{2}\left(\frac{1}{\cos\alpha }-1\right)F_{b}^{nl}\right] \end{array}$$

(36) $$\frac{dM_{n}^{nl}}{d\theta }=\sin\alpha M_{b}^{nl}-\cos\alpha M_{t}^{nl}+\frac{R}{\cos\alpha }F_{b}^{nl}$$

(37) $$\frac{dM_{b}^{nl}}{d\theta }=-\sin\alpha M_{n}^{nl}-\frac{R}{\cos\alpha }F_{n}^{nl}$$

(38) $$\frac{dM_{t}^{nl}}{d\theta }=\cos\alpha M_{n}^{nl}$$

(39) $$\frac{dF_{n}^{nl}}{d\theta }=\sin\alpha F_{b}^{nl}-\cos\alpha F_{t}^{nl}$$

(40) $$\frac{dF_{b}^{nl}}{d\theta }=-\sin\alpha F_{n}^{nl}$$

(41) $$\frac{dF_{t}^{nl}}{d\theta }=\cos\alpha F_{n}^{nl}$$

where $u_{n}^{nl}$, $u_{b}^{nl}$, and $u_{t}^{nl}$ denote displacements, $\Omega_{n}^{nl}$, $\Omega_{b}^{nl}$, and $\Omega_{t}^{nl}$ are rotations, A is the cross-sectional area E and G are the Young’s and shear moduli, respectively; $I_{n}$, and $I_{b}$ are the second moments of area with respect to n and b axes; J is the torsional constant of the cross-section.

## 3. Exact Analytical Solution by Using the Initial Value Method

The solution procedure follows a structured sequence. First, the force resultants are obtained from the equilibrium relations. These are then used to determine the internal moments. Subsequently, the rotations are computed from the moment–curvature relations, and finally the displacements are recovered from the rotation–displacement kinematics. This stepwise progression ensures that the governing system can be solved analytically in a transparent and reproducible manner.

From this stage, the equations are presented in matrix form, where a linear first-order constant coefficient ODE system is expressed as:

(42) $$\frac{dy(\theta )}{d\theta }=Ay(\theta )+f\left(\theta \right)$$

Here, $y\left(\theta \right)$ denotes the state vector of system variables, A is the constant coefficient matrix arising from geometric uniformity (constant radius, pitch angle, and cross-section), and $f\left(\theta \right)$ represents the nonhomogeneous vector. The general solution of Equation (42) is well known and can be written as:

(43) $$y\left(\theta \right)=Y\left(\theta ,\theta_{0}\right)y_{0}+Y\left(\theta ,\theta_{0}\right)\int_{\theta_{0}}^{\theta }Y^{-1}\left(\xi ,x_{0}\right)f\left(\xi \right)d\xi$$

The expression is valid provided that the initial state $y_{0}=y(\theta_{0})$ is known. The fundamental matrix. $Y\left(\theta ,\theta_{0}\right)$, defined relative to the reference coordinate $\theta_{0}$, is derived from the homogeneous counterpart of Equation (42):

(44) $$y\left(\theta \right)=Y\left(\theta ,\theta_{0}\right)y_{0}$$

The fundamental matrix $Y\left(\theta ,\theta_{0}\right)$ satisfies the following relations:

(45) $$\frac{dY\left(\theta ,\theta_{0}\right)}{d\theta }=AY\left(\theta ,\theta_{0}\right)$$

(46) $$Y\left(\theta_{0},\theta_{0}\right)=I$$

(47) $$Y^{-1}\left(\theta_{1},\theta_{2}\right)=Y\left(\theta_{2},\theta_{1}\right)$$

(48) $$Y\left(\theta_{1},\theta_{2}\right)Y\left(\theta_{2},\theta_{3}\right)=Y\left(\theta_{1},\theta_{3}\right)$$

Here, I denotes the identity matrix. Equations (30)–(41) form a linear first-order homogeneous system of ordinary differential equations, solved through the initial value method. The reference coordinate is taken as $\theta_{0}$ = 0, so that $y_{0}=y\left(0\right)$.

As a first step, Equations (39)–(41) for the force resultants define a linear first-order homogeneous system of differential equations:

(49) $$\frac{d}{d\theta }\left[\begin{array}{c} F_{n}^{nl}\\ F_{b}^{nl}\\ F_{t}^{nl} \end{array}\right]=\left[\begin{array}{ccc} 0 & \sin\alpha & -\cos\alpha \\ -\sin\alpha & 0 & 0\\ \cos\alpha & 0 & 0 \end{array}\right]\left[\begin{array}{c} F_{n}^{nl}\\ F_{b}^{nl}\\ F_{t}^{nl} \end{array}\right]$$

Equation (49) corresponds to the force-resultant subsystem of the helical beam. They describe how the internal forces vary along the arc length of the helix under the imposed loading and boundary conditions. Solving this subsystem provides the foundation for determining the subsequent moment and rotation fields.

The expressions for $F_{n}^{nl}$, $F_{b}^{nl}$, and $F_{t}^{nl}$ are analytically obtained as:

(50) $$\left[\begin{array}{c} F_{n}^{nl}\\ F_{b}^{nl}\\ F_{t}^{nl} \end{array}\right]=Y\left(\theta ,0\right)\left[\begin{array}{c} F_{n0}^{nl}\\ F_{b0}^{nl}\\ F_{t0}^{nl} \end{array}\right]$$

Hence, the fundamental matrix $Y\left(\theta ,0\right)$ reads:

(51) $$Y\left(\theta ,0\right)=\left[\begin{array}{ccc} \cos\theta & \sin\alpha \sin\theta & -\cos\alpha \sin\theta \\ -\sin\alpha \sin\theta & \cos^{2}\alpha +\sin^{2}\alpha \cos\theta & \sin\alpha \cos\alpha \left(1-\cos\theta \right)\\ \cos\alpha \sin\theta & \sin\alpha \cos\alpha \left(1-\cos\theta \right) & \sin^{2}\alpha +\cos^{2}\alpha \cos\theta \end{array}\right]$$

Next, considering the moment relations in Equations (36)–(38) reveals that they form a non-homogeneous system. The coefficient matrix A associated with their homogeneous component is the same as that defined in Equation (49):

(52) $$\frac{d}{d\theta }\left[\begin{array}{c} M_{n}^{nl}\\ M_{b}^{nl}\\ M_{t}^{nl} \end{array}\right]=\left[\begin{array}{ccc} 0 & \sin\alpha & -\cos\alpha \\ -\sin\alpha & 0 & 0\\ \cos\alpha & 0 & 0 \end{array}\right]\left[\begin{array}{c} M_{n}^{nl}\\ M_{b}^{nl}\\ M_{t}^{nl} \end{array}\right]+\left[\begin{array}{c} \frac{R}{\cos\alpha }F_{b}^{nl}\\ -\frac{R}{\cos\alpha }F_{n}^{nl}\\ 0 \end{array}\right]$$

The moment subsystem inherits the same coefficient matrix as the force subsystem, meaning that the same fundamental matrix can be reused. This structural similarity reduces computational effort and highlights the close coupling between internal forces and bending/torsional moments in the helical geometry.

Accordingly, the fundamental matrix is the same as in Equation (50). Advancing to the particular solution requires determining the inverse $Y^{-1}\left(\theta ,0\right)$, which is necessary for computing the nonhomogeneous contribution in Equation (43).

(53) $$Y^{-1}\left(\theta ,0\right)=\left[\begin{array}{ccc} \cos\theta & -\sin\alpha \sin\theta & \cos\alpha \sin\theta \\ \sin\alpha \sin\theta & \cos^{2}\alpha +\sin^{2}\alpha \cos\theta & \sin\alpha \cos\alpha \left(1-\cos\theta \right)\\ -\cos\alpha \sin\theta & \sin\alpha \cos\alpha \left(1-\cos\theta \right) & \sin^{2}\alpha +\cos^{2}\alpha \cos\theta \end{array}\right]$$

Afterwards, since $F_{n}^{nl}$ and $F_{b}^{nl}$ in the nonhomogeneous part of Equation (51) are known, the solution of the system can be written as:

(54) $$\left[\begin{array}{c} M_{n}^{nl}\\ M_{b}^{nl}\\ M_{t}^{nl} \end{array}\right]=Y\left(\theta ,0\right)\left[\begin{array}{c} M_{n0}^{nl}\\ M_{b0}^{nl}\\ M_{t0}^{nl} \end{array}\right]+Y\left(\theta ,0\right)\int_{0}^{\theta }Y^{-1}\left(\xi ,0\right)\left[\begin{array}{c} \frac{R}{\cos\alpha }F_{b}^{nl}\\ -\frac{R}{\cos\alpha }F_{n}^{nl}\\ 0 \end{array}\right]d\xi$$

Equations (33)–(35), which describe the cross-sectional rotation angles of the beam, can be expressed in matrix form as:

(55) $$\begin{array}{c} \frac{d}{d\theta }\left[\begin{array}{c} \Omega_{n}^{nl}\\ \Omega_{b}^{nl}\\ \Omega_{t}^{nl} \end{array}\right]\\ =\left[\begin{array}{ccc} 0 & \sin\alpha & -\cos\alpha \\ -\sin\alpha & 0 & 0\\ \cos\alpha & 0 & 0 \end{array}\right]\left[\begin{array}{c} \Omega_{n}^{nl}\\ \Omega_{b}^{nl}\\ \Omega_{t}^{nl} \end{array}\right]\\ +\left[\begin{array}{c} \frac{R}{\cos\alpha EI_{n}}\left(1+\frac{\gamma^{2}}{R^{2}}\right)M_{n}^{nl}+\frac{\gamma^{2}}{R^{2}}\frac{2R^{2}\sin\alpha }{EI_{n}{\left(\cos\alpha \right)}^{2}}F_{n}^{nl}\\ \frac{R}{\cos\alpha EI_{b}}\left[\left(1+\frac{\gamma^{2}}{R^{2}}{\left(\sin\alpha \right)}^{2}\right)M_{b}^{nl}-\frac{\gamma^{2}}{R^{2}}\sin\alpha \cos\alpha M_{t}^{nl}+2\frac{\gamma^{2}}{R^{2}}\frac{\sin\alpha }{\cos\alpha }RF_{b}^{nl}\right]\\ \frac{R}{\cos\alpha GJ}\left[\left(1+\frac{\gamma^{2}}{R^{2}}\left(1+{\left(\cos\alpha \right)}^{2}-\frac{\cos\alpha }{2}\right)\right)M_{t}^{nl}+\frac{\gamma^{2}}{R^{2}}\sin\alpha \left(\frac{1}{2}-\cos\alpha \right)M_{b}^{nl}+\frac{\gamma^{2}}{2R}\left(\frac{1}{\cos\alpha }-1\right)F_{b}^{nl}\right] \end{array}\right] \end{array}$$

The rotation equations link the internal moments directly to the kinematic response of the cross-section. Once the rotations are determined, the displacement field can be constructed, completing the chain from equilibrium to geometry.

Since the homogeneous portion of the equation shares the same coefficient matrix A as Equations (49) and (52), the associated fundamental matrix $Y\left(\theta ,0\right)$ and its inverse $Y^{-1}\left(\theta ,0\right)$ coincide with those in Equations (51) and (53). Hence, the rotations relative to the Frenet coordinate system are determined by:

(56) $$\begin{array}{c} \left[\begin{array}{c} \Omega_{n}^{nl}\\ \Omega_{b}^{nl}\\ \Omega_{t}^{nl} \end{array}\right]\\ =Y\left(\theta ,0\right)\left[\begin{array}{c} \Omega_{n0}^{nl}\\ \Omega_{b0}^{nl}\\ \Omega_{t0}^{nl} \end{array}\right]\\ +Y\left(\theta ,0\right)\int_{0}^{\theta }Y^{-1}\left(\xi ,0\right)\left[\begin{array}{c} \frac{R}{\cos\alpha EI_{n}}\left(1+\frac{\gamma^{2}}{R^{2}}\right)M_{n}^{nl}+\frac{\gamma^{2}}{R^{2}}\frac{2R^{2}\sin\alpha }{EI_{n}{\left(\cos\alpha \right)}^{2}}F_{n}^{nl}\\ \frac{R}{\cos\alpha \;EI_{b}}\left[\left(1+\frac{\gamma^{2}}{R^{2}}{\left(\sin\alpha \right)}^{2}\right)M_{b}^{nl}-\frac{\gamma^{2}}{R^{2}}\sin\alpha \cos\alpha M_{t}^{nl}+2\frac{\gamma^{2}}{R}\frac{\sin\alpha }{\cos\alpha }F_{b}^{nl}\right]\\ \frac{R}{\cos\alpha GJ}\left[\left(1+\frac{\gamma^{2}}{R^{2}}\left(1+{\left(\cos\alpha \right)}^{2}-\frac{\cos\alpha }{2}\right)\right)M_{t}^{nl}+\frac{\gamma^{2}}{R^{2}}\sin\alpha \left(\frac{1}{2}-\cos\alpha \right)M_{b}^{nl}+\frac{\gamma^{2}}{2R}\left(\frac{1}{\cos\alpha }-1\right)F_{b}^{nl}\right] \end{array}\right]d\xi \end{array}$$

Here, the internal moments $M_{n}^{nl}$, $M_{b}^{nl}$, and $M_{t}^{nl}$, and the forces $F_{n}^{nl}$, and $F_{b}^{nl}$ are defined in Equations (50) and (54).

(57) $$\begin{array}{c} \frac{d}{d\theta }\left[\begin{array}{c} u_{n}^{nl}\\ u_{b}^{nl}\\ u_{t}^{nl} \end{array}\right]\\ =\left[\begin{array}{ccc} 0 & \sin\alpha & -\cos\alpha \\ -\sin\alpha & 0 & 0\\ \cos\alpha & 0 & 0 \end{array}\right]\left[\begin{array}{c} u_{n}^{nl}\\ u_{b}^{nl}\\ u_{t}^{nl} \end{array}\right]\\ +\left[\begin{array}{c} \frac{R}{\cos\alpha }\Omega_{b}^{nl}+\frac{k_{n}R}{\cos\alpha GA}\left(1+\frac{\gamma^{2}}{R^{2}}\right)F_{n}^{nl}\\ -\frac{R}{\cos\alpha }\Omega_{n}^{nl}+\frac{k_{b}R}{\cos\alpha GA}\left(1+\frac{\gamma^{2}}{R^{2}}\left[1+{\left(\sin\alpha \right)}^{2}\right]\right)F_{b}^{nl}-\frac{k_{b}R}{GA}\frac{\gamma^{2}}{R^{2}}\sin\alpha F_{t}^{nl}\\ \frac{R}{\cos\alpha EA}\left(1+\frac{\gamma^{2}}{R^{2}}{\left(\cos\alpha \right)}^{2}\right)F_{t}^{nl}-\frac{\gamma^{2}}{R^{2}}\frac{R}{EA}\sin\alpha F_{b}^{nl} \end{array}\right] \end{array}$$

Accordingly, the displacement components can be expressed as:

(58) $$\begin{array}{c} \left[\begin{array}{c} u_{n}^{nl}\\ u_{b}^{nl}\\ u_{t}^{nl} \end{array}\right]\\ =Y\left(\theta ,0\right)\left[\begin{array}{c} u_{n0}^{nl}\\ u_{b0}^{nl}\\ u_{t0}^{nl} \end{array}\right]\\ +Y\left(\theta ,0\right)\int_{0}^{\theta }Y^{-1}\left(\xi ,0\right)d\xi \left[\begin{array}{c} \frac{R}{\cos\alpha }\Omega_{b}^{nl}+\frac{k_{n}R}{\cos\alpha GA}\left(1+\frac{\gamma^{2}}{R^{2}}\right)F_{n}^{nl}\\ -\frac{R}{\cos\alpha }\Omega_{n}^{nl}+\frac{k_{b}R}{\cos\alpha GA}\left(1+\frac{\gamma^{2}}{R^{2}}\left[1+{\left(\sin\alpha \right)}^{2}\right]\right)F_{b}^{nl}-\frac{k_{b}R}{GA}\left(\frac{\gamma^{2}}{R^{2}}\sin\alpha F_{t}^{nl}\right)\\ \frac{R}{\cos\alpha EA}\left(1+\frac{\gamma^{2}}{R^{2}}{\left(\cos\alpha \right)}^{2}\right)F_{t}^{nl}-\frac{\gamma^{2}}{R^{2}}\frac{R}{EA}\sin\alpha F_{b}^{nl} \end{array}\right] \end{array}$$

Here, $F_{n}^{nl}$, $F_{b}^{nl}$, and $F_{t}^{nl}$ are given by Equation (38), while $\Omega_{n}^{nl}$, and $\Omega_{b}^{nl}$ are obtained from Equation (56).

The overall analytical solution can therefore be explained as a pipeline: Forces → Moments → Rotations → Displacements. Each stage builds on the previous one, with the fundamental matrix providing a unifying tool to solve the coupled ordinary differential equations.

By solving the force, moment, rotation, and displacement equations sequentially through the initial value method, the complete analytical solution of the static behaviour of helical SWCNT is obtained. This yields closed-form relations for all quantities as functions of the helix angle θ. Due to the algebraic complexity of the general solution, only a representative case is provided here: the closed-coil SWCNT (tension spring) with a pitch angle approaching zero (α≈0), for which the analytical results are:

(59) $$\begin{array}{c} u_{n}^{nl}\left(\theta \right)=u_{n0}^{nl}\cos\theta -u_{t0}^{nl}\sin\theta +\Omega_{b0}^{nl}R\sin\theta +M_{b0}^{nl}\frac{R^{2}}{EI_{b}}\left(1-\cos\theta \right)\\ +F_{t0}^{nl}\left[\frac{R^{3}}{EI_{b}}\left(1-\cos\theta -\frac{\theta \sin\theta }{2}\right)\right]\\ +F_{n0}^{nl}\left[\frac{R^{3}}{2EI_{b}}\left(\theta \cos\theta -\sin\theta \right)\right] \end{array}$$

(60) $$\begin{array}{cc} u_{b}^{nl}\left(\theta \right)=u_{b0}^{nl}- & \Omega_{n0}^{nl}R\sin\theta +\Omega_{t0}^{nl}R\left(1-\cos\theta \right)\\ & +M_{n0}^{nl}[\frac{R^{2}}{GJ}\left(1+\frac{3\gamma^{2}}{2R^{2}}\right)\left(1-\cos\theta -\frac{\theta \sin\theta }{2}\right)\\ & -\frac{R^{2}}{2EI_{n}}\left(1+\frac{\gamma^{2}}{R^{2}}\right)\theta \sin\theta ]\\ & +M_{t0}^{nl}[\frac{R^{2}}{2EI_{n}}\left(1+\frac{\gamma^{2}}{R^{2}}\right)\left(\sin\theta -\theta \cos\theta \right)\\ & +\frac{R^{2}}{2GJ}\left(1+\frac{3\gamma^{2}}{2R^{2}}\right)\left(\sin\theta -\theta \cos\theta \right)]\\ & +F_{b0}^{nl}[\frac{R^{3}}{2EI_{n}}\left(1+\frac{\gamma^{2}}{R^{2}}\right)\left(\theta \cos\theta -\sin\theta \right)\\ & +\frac{R^{3}}{2GJ}\left(1+\frac{3\gamma^{2}}{2R^{2}}\right)\left(2\theta +\theta \cos\theta -3\sin\theta \right)] \end{array}$$

(61) $$\begin{array}{cc} u_{t}^{nl}\left(\theta \right)=u_{n0}^{nl}\sin\theta & +u_{t0}^{nl}\cos\theta +\Omega_{b0}^{nl}R\left(1-\cos\theta \right)+M_{b0}^{nl}\frac{R^{2}}{EI_{b}}\left(\theta -\sin\theta \right)\\ & +F_{n0}^{nl}\left[\frac{R^{3}}{EI_{b}}\left(\cos\theta -1+\frac{\theta }{2}\sin\theta \right)\right]\\ & +F_{t0}^{nl}\left[\frac{R^{3}}{EI_{b}}\left(\theta -\frac{3}{2}\sin\theta +\frac{\theta }{2}\cos\theta \right)\right] \end{array}$$

(62) $$\begin{array}{cc} \Omega_{n}^{nl}\left(\theta \right)=\Omega_{n0}^{nl}\cos\theta & -\;\Omega_{t0}^{nl}\sin\theta \\ & +\;M_{n0}^{nl}\frac{R}{2}[\frac{1}{EI_{n}}\left(1+\frac{\gamma^{2}}{R^{2}}\right)\left(\theta \cos\theta +\sin\theta \right)\\ & +\;\frac{1}{GJ}\left(1+\frac{3\gamma^{2}}{2R^{2}}\right)\left(\theta \cos\theta -\sin\theta \right)]\\ & +\;M_{t0}^{nl}\frac{R}{2}\theta \sin\theta \left[-\frac{1}{EI_{n}}\left(1+\frac{\gamma^{2}}{R^{2}}\right)-\frac{1}{GJ}\left(1+\frac{3\gamma^{2}}{2R^{2}}\right)\right]\\ & +\;F_{b0}^{nl}\frac{R^{2}}{2}[\frac{1}{EI_{n}}\left(1+\frac{\gamma^{2}}{R^{2}}\right)\theta \sin\theta \\ & -\;\frac{1}{GJ}\left(1+\frac{3\gamma^{2}}{2R^{2}}\right)\left(2-\theta \sin\theta -2\cos\theta \right)] \end{array}$$

(63) $$\Omega_{b}^{nl}\left(\theta \right)=\Omega_{b0}^{nl}+M_{b0}^{nl}\frac{R\theta }{EI_{b}}-F_{n0}^{nl}\frac{R^{2}}{EI_{b}}\left(1-\cos\theta \right)+F_{t0}^{nl}\frac{R^{2}}{EI_{b}}\left(\theta -\sin\theta \right)$$

(64) $$\begin{array}{cc} \Omega_{t}^{nl}\left(\theta \right)=\Omega_{t0}^{nl}\cos\theta & +\Omega_{n0}^{nl}\sin\theta +M_{n0}^{nl}\left[\frac{1}{EI_{n}}\left(1+\frac{\gamma^{2}}{R^{2}}\right)+\frac{1}{GJ}\left(1+\frac{3\gamma^{2}}{2R^{2}}\right)\right]\frac{R\theta }{2}\sin\theta \\ & +M_{t0}^{nl}\left[\frac{1}{EI_{n}}\left(1+\frac{\gamma^{2}}{R^{2}}\right)+\frac{1}{GJ}\left(1+\frac{3\gamma^{2}}{2R^{2}}\right)\right]\frac{R}{2}\left(\theta \cos\theta -\sin\theta \right)\\ & +F_{b0}^{nl}\left[\frac{1}{EI_{n}}\left(1+\frac{\gamma^{2}}{R^{2}}\right)+\frac{1}{GJ}\left(1+\frac{3\gamma^{2}}{2R^{2}}\right)\right]\frac{R^{2}}{2}\left(\sin\theta -\theta \cos\theta \right) \end{array}$$

(65) $$M_{n}^{nl}\left(\theta \right)=M_{n0}^{nl}\cos\theta -M_{t0}^{nl}\sin\theta +F_{b0}^{nl}R\sin\theta$$

(66) $$M_{b}^{nl}\left(\theta \right)=M_{b0}^{nl}+F_{t0}^{nl}R\left(1-\cos\theta \right)-F_{n0}^{nl}R\sin\theta$$

(67) $$M_{t}^{nl}\left(\theta \right)=M_{t0}^{nl}\cos\theta +M_{n0}^{nl}\sin\theta +F_{b0}^{nl}R\left(1-\cos\theta \right)$$

(68) $$F_{n}^{nl}\left(\theta \right)=F_{n0}^{nl}\cos\theta -F_{t0}^{nl}\sin\theta$$

(69) $$F_{b}^{nl}\left(\theta \right)=F_{b0}^{nl}$$

(70) $$F_{t}^{nl}\left(\theta \right)=F_{t0}^{nl}\cos\theta +F_{n0}^{nl}\sin\theta$$

## 4. Numerical Examples

An analytical solution can be efficiently obtained, provided that the initial values are known. These initial values can be determined by solving a system of linear equations derived directly from the boundary conditions of the beam.

To demonstrate the application of the analytical solution procedure, two cases based on two sets of boundary and loading conditions are examined.

1. Case 1: Clamped–Free Beam with a Concentrated Load at the Free End

A helical beam fixed at one end, A, and free at the other, B, is analysed. At the free end, concentrated forces ($F_{nB}$, $F_{bB}$, and $F_{tB}$) and moments ($M_{nB}$, $M_{bB}$, and $M_{tB}$) are applied individually to study the response of the beam. The corresponding initial value vector at θ=0, at end A, is constructed by enforcing zero displacements and rotations at the clamped end and assigning the appropriate force or moment at the free end $\theta =\theta_{B}$.

The clamped boundary conditions are given for the end A of the beam:

(71) $$u_{nA}=0,\;\;\;u_{bA}=0,\;\;\;\;\;\;u_{tA}=0,\;\;\;\;\;\;\;\Omega_{nA}=0,\;\;\;\;\;\;\;\Omega_{bA}=0,\;\;\;\;\;\;\;\Omega_{tA}=0$$

When the loads are applied to the free end B, the related equations must be equal to the loads.

(72) $$F_{nB}=0,\;{\;\;F}_{bB}=F_{z}\cos\alpha ,\;\;F_{tB}=F_{z}\sin\alpha ,\;\;M_{nB}=0,\;\;\;\;M_{bB}=0,\;\;\;M_{tB}=0$$

Substituting these conditions into the governing Equations (50), (54), (56) and (58) provides the distributions of resultants, displacements, and rotations along the beam.

2. Case 2: Clamped–Clamped Beam with a Concentrated Load at an Intermediate Point

The second configuration considers a beam clamped at both ends and subjected to concentrated loads at a point $\theta =\theta_{C}$. Each load case is again applied individually for $F_{nC}$, $F_{bC}$, $F_{tC}$, $M_{nC}$, $M_{bC}$, and $M_{tC}$. The problem is divided into two subdomains (before and after the loading point), with continuity of displacements, rotations and force and moment resultants enforced at the load application point. The load introduces a discontinuity in the internal force or moment vector, which is accounted for through the continuity conditions. The clamped boundary conditions at end A, θ=0 and at end B, $\theta =\theta_{B}\;$ are imposed to determine the full initial value vector.

When a beam is subjected to point loads $M_{nC},M_{bC},M_{tC},F_{nC},F_{bC},$ and $F_{tC}$ at a specific coordinate $\theta =\theta_{C}$, the domain is divided into two subregions, each governed by its corresponding analytical solution:

(73) $$y_{1}\left(\theta_{1}\right)=Y\left(\theta_{1},0\right)y_{10}\;\;\;\;\;\;\;\;\;\;\;for\;\;\;\;\;\;0<\theta_{1}<\theta_{C}$$

(74) $$y_{2}\left(\theta_{2}\right)=Y\left(\theta_{2},\theta_{C}\right)y_{2C}\;\;\;\;\;\;\;\;\;for\;\;\;\;\;\;\theta_{C}<\theta_{1}<\theta_{t}$$

Here, $\theta_{t}$ is the total helix angle, $y_{2C}$ is the initial value at coordinate $\theta =\theta_{C}$ for the second region. To ensure continuity at this interface, the following condition is imposed:

(75) $$y_{2C}=y_{1}\left(\theta_{C}\right)+K$$

where K is the external loading vector, defined as:

(76) $$K={\left[0,\;0,\;0,\;0,\;0,\;0,M_{nC},M_{bC},M_{tC},F_{nC},F_{bC},F_{tC}\right]}^{T}$$

Substituting this into the governing expression yields:

(77) $$y_{2}\left(\theta_{2}\right)=Y\left(\theta_{2},\theta_{C}\right)y_{1}\left(\theta_{C}\right)+Y\left(\theta_{2},\theta_{C}\right)K$$

(78) $$y_{2}\left(\theta_{2}\right)=Y\left(\theta_{2},\theta_{C}\right)Y\left(\theta_{C},0\right)y_{10}+Y\left(\theta_{2},\theta_{C}\right)K$$

(79) $$y_{2}\left(\theta_{2}\right)=Y\left(\theta_{2},0\right)y_{10}+Y\left(\theta_{2},0\right)Y^{-1}\left(\theta_{C},0\right)K$$

Once the initial state at θ=0 is known, the displacements and rotations at any location along the helical beam subjected to a concentrated load can be determined by solving the governing differential equations, such as Equations (49), (52), (55) and (57).

## 5. Results and Discussions of the Numerical Examples

In order to simplify and parameterise the problem, the formulation for helical beams with different pitch angles, α, aspect ratio, R/d, and winding angle, θ is nondimensionalised. Therefore, the force to be applied is associated with beam geometry and the resulting displacement values become more consistent. The effects of the winding angle, θ, pitch angle, α, and aspect ratio, R/d, on the displacement at the free end are investigated. The aspect ratio is R/d = 2.5, and the nondimensional force is $\bar{F}=c^{2}F/\left(EI_{n}\right)=10^{-3}$. Nondimensional displacements are defined as $\bar{u}=u/c$, Poisson’s ratio ν = 0.3. This nondimensionalization allows the deformation behaviour to be expressed in terms of geometric ratios and intrinsic material properties, enabling more efficient parametric studies across a wide design space.

Table 1 summarises the normalised displacement and rotation responses of cantilevered helical SWCNT beams subjected to end-applied axial loads, comparing local and nonlocal elasticity predictions for different winding angles (θ = 4π, 10π), pitch angles (α = 5°, 10°, 15°), and nonlocal parameters (R/γ = 1, 5, 10).

The results confirm that the nonlocal parameter strongly influences the deformation. At small R/γ (e.g., 1), nonlocal predictions deviate significantly from the local model. For θ = 4π and α = 5°, the normal displacement ${\bar{u}}_{n}$ is −0.447169 compared to −0.270136 locally, an increase of about 65%. As R/γ increases to 10, results approach the local solution, reflecting the diminishing role of nonlocal effects when the characteristic length scale is small relative to beam dimensions.

The winding angle also affects stiffness: beams with larger θ (10π) exhibit smaller axial and tangential displacements than those with θ = 4π, consistent with increased effective length and reinforcement from additional turns. The pitch angle modifies this trend at α = 5° displacements along the normal axis are negative, whereas at α = 15° they become positive in several cases (e.g., ${\bar{u}}_{n}$ = 1.141435 for θ = 4π, R/γ = 1), showing that pitch can redirect deformation and even lead to net elongation.

Tangential displacements ${\bar{u}}_{t}$ are particularly sensitive to both α and R/γ, with some cases reversing sign relative to the local model, reflecting coupling between curvature and torsion. The bending displacement ${\bar{u}}_{b}$ also exhibits sign changes, indicating complex geometric sensitivity.

Rotational responses amplify under nonlocal conditions. For instance, at θ = 10π, α = 15°, and R/γ = 1, the bending rotation $\Omega_{b}$ reaches 0.556787—over twenty times the local prediction (0.025739). Torsional rotations $\Omega_{t}$ similarly show pronounced amplification and geometry dependent sign changes.

**Figure 2:**
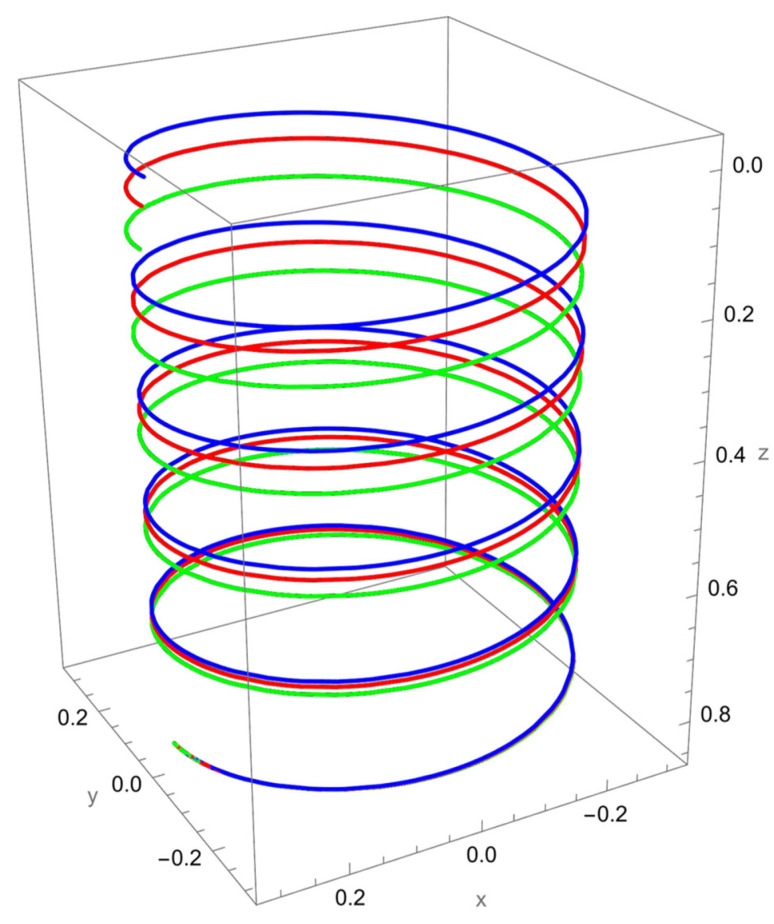
A cantilevered helical SWCNT beam under a vertical axial force applied along the cylindrical axis. Blue denotes the undeformed axis, red indicates the displacement predicted by local elasticity, and green shows the result of the nonlocal elasticity theory.

As shown in Figure 2, when a vertical axial force is applied at the free end of the cantilevered helical SWCNT, both local and nonlocal elasticity models predict elongation of the helix along the cylindrical axis, but with notable differences. The nonlocal solution consistently yields larger axial displacements compared to the local model, reflecting the characteristic softening effect introduced by nanoscale interactions.

The discrepancy between local and nonlocal predictions is particularly significant when the nonlocal parameter R/γ is small, since the internal characteristic length is then comparable to the beam’s geometry. As R/γ increases, the two solutions converge, indicating a reduced influence of nonlocality.

Geometric effects also play a role: the helical winding and pitch angles couple the applied axial load into lateral displacements, leading to small deviations from the cylinder axis. These deviations are more pronounced in the nonlocal predictions, showing that curvature–torsion interactions are magnified when size effects are included.

**Figure 3:**
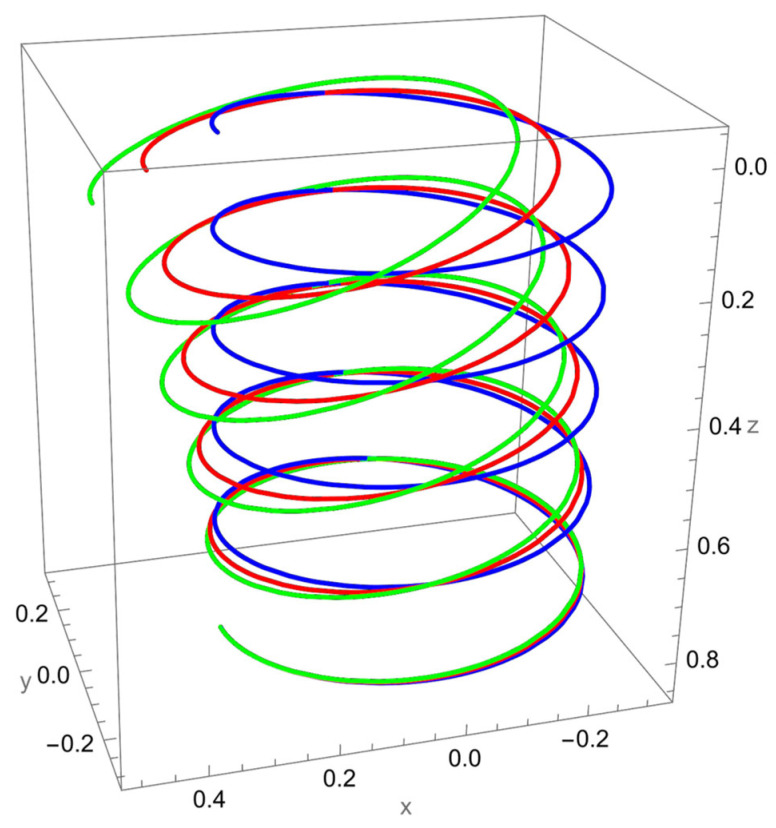
Deformed configuration of a cantilevered helical SWCNT beam subjected to a force $F_{n}$ applied at the free end along the normal axis. The blue line indicates the undeformed configuration, the red curve represents the deformation predicted by classical (local) elasticity theory, and the green curve illustrates the displacement response obtained using nonlocal elasticity theory.

In Figure 3, the response to a concentrated normal force reveals the strong influence of nonlocal elasticity on bending behaviour. Both local and nonlocal elasticity models capture this bending trend, but the magnitude of the response differs significantly.

The nonlocal solution consistently predicts larger lateral displacements than the local model. This amplification reflects the scale-dependent softening effect, where interatomic interactions reduce the effective bending stiffness. The difference is particularly evident for smaller values of the nonlocal parameter R/γ, with convergence toward the local prediction as R/γ increases.

In addition to the increased lateral deflection, the helical geometry introduces coupling between bending and torsion. This coupling causes secondary displacements along the tangential and axial directions, visible in the green and red curves. The nonlocal predictions show more pronounced coupling effects, confirming that nanoscale interactions enhance geometric sensitivity.

**Figure 4:**
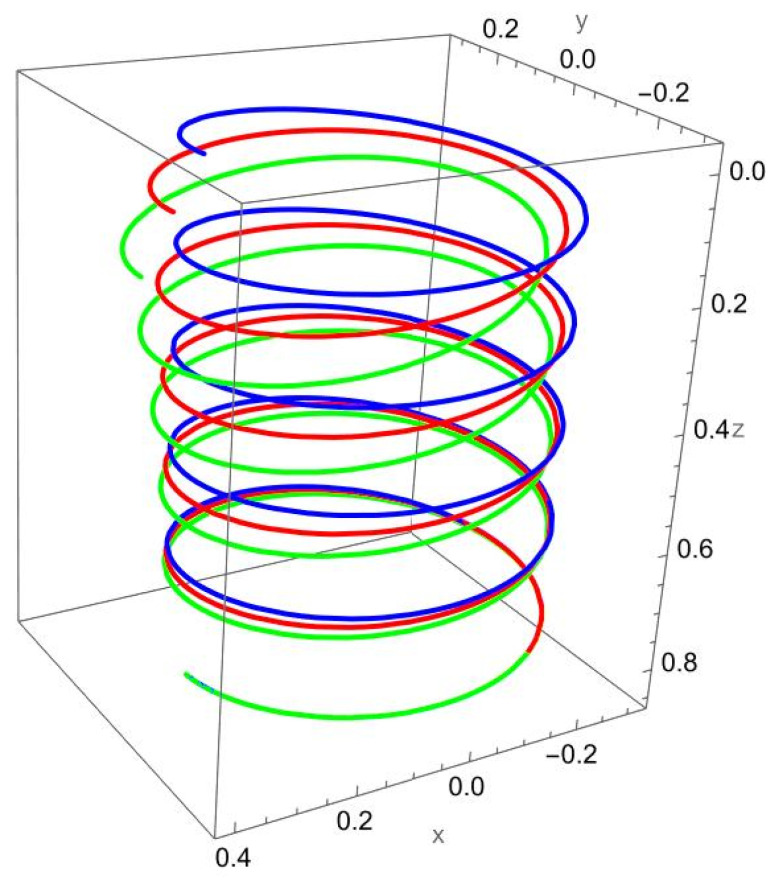
A cantilevered helical SWCNT beam under a concentrated binormal force $F_{b}$ applied at the free end. Blue denotes the undeformed axis, red indicates the displacement predicted by local elasticity, and green shows the result of the nonlocal elasticity theory.

Figure 4 illustrates the deformation of the cantilevered helical SWCNT beam under a binormal force applied at the free end. The dominant response is lateral bending in the binormal direction, with both local and nonlocal models capturing the overall shape of the deformation. However, as in previous cases, the magnitude of displacement differs: the nonlocal solution predicts noticeably larger deflections compared to the local elasticity model.

The discrepancy between the two theories is most evident at low R/γ values, where nonlocal effects are strongest. As R/γ increases, the curves predicted by the two models begin to converge, confirming that the influence of scale-dependent interactions diminishes when the internal length scale is small relative to the structural dimensions.

The helical geometry also leads to coupling between binormal bending and other displacement components. The applied $F_{b}$ not only induces lateral deflection but also results in tangential shifts and small axial displacements. These coupled responses are more pronounced in the nonlocal predictions, reflecting the enhanced sensitivity of nanoscale helices to load transfer across curved geometries.

**Figure 5:**
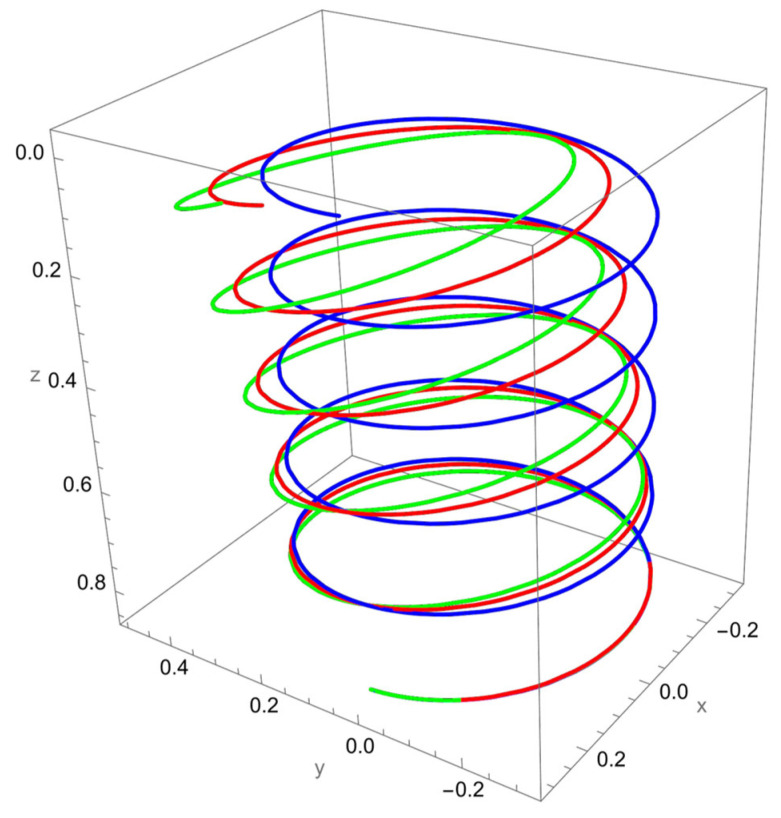
A cantilevered helical SWCNT beam under a concentrated tangential force $F_{t}$ applied at the free end. Blue denotes the undeformed axis, red indicates the displacement predicted by local elasticity, and green shows the result of the nonlocal elasticity theory.

Figure 5 shows the deformation of the cantilevered helical SWCNT beam subjected to a tangential force applied at the free end. Unlike axial or binormal loading, tangential excitation directly drives torsional deformation, which is strongly coupled with bending due to the helical geometry. Both the local and nonlocal predictions exhibit a pronounced twisting response along the cylindrical axis, but the nonlocal theory predicts consistently larger displacements and rotations.

The difference between local and nonlocal solutions is particularly evident in the lateral spread of the helix. For small R/γ, the green curve departs significantly from the red curve, highlighting the scale-dependent reduction in torsional stiffness. As R/γ increases, the discrepancy decreases, with the two predictions converging towards each other.

Coupling effects are especially important under tangential loading. The torsional excitation induces not only twisting about the axis but also secondary normal and binormal displacements, which are more pronounced in the nonlocal model. This reflects the fact that nanoscale interactions amplify cross-coupling between torsion and bending in curved structures.

**Figure 6:**
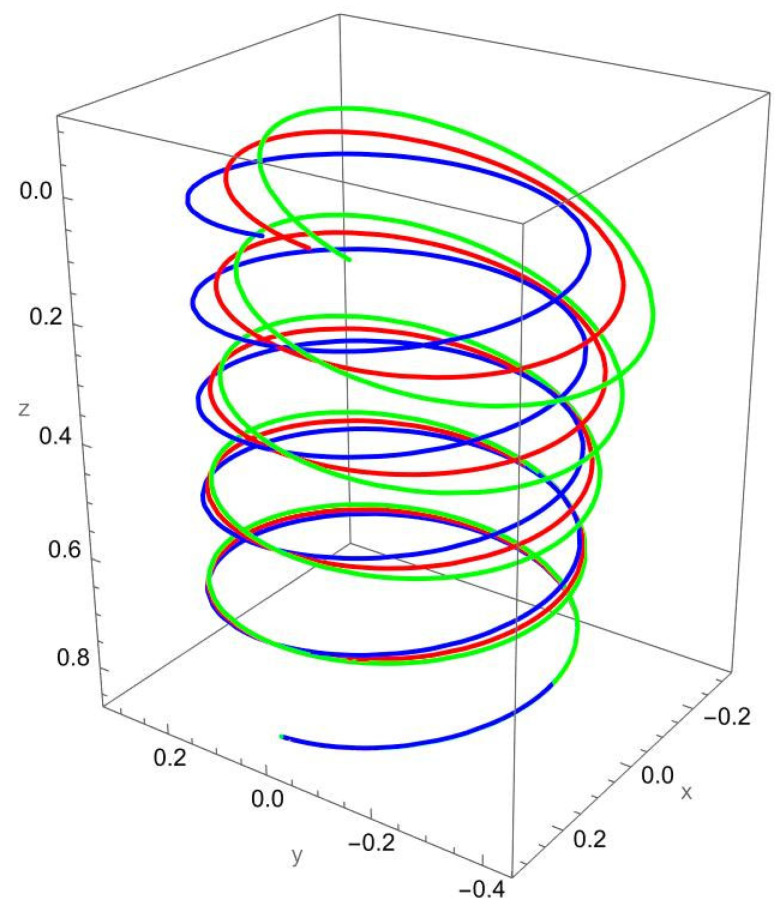
A cantilevered helical SWCNT beam under a concentrated normal moment $M_{n}$ applied at the free end. Blue denotes the undeformed axis, red indicates the displacement predicted by local elasticity, and green shows the result of the nonlocal elasticity theory.

Figure 6 illustrates the response of the cantilevered helical SWCNT beam subjected to a normal moment at the free end. This loading primarily induces bending about the normal axis, producing a rotational deformation that propagates along the helix. Both local and nonlocal theories predict similar qualitative behaviour, but the magnitude of displacement and rotation is markedly higher in the nonlocal case.

The difference between the two models reflects the reduced bending stiffness captured by nonlocal elasticity. For small values of the nonlocal parameter R/γ, the green curve departs significantly from the red, with amplified deflections and rotations. As R/γ increases, the predictions converge, showing that the impact of size effects diminishes when the internal length scale is small relative to the helical geometry.

An important feature of this loading case is the coupling between bending and torsional responses. The applied normal moment not only generates curvature in the normal plane but also induces tangential and binormal displacements due to the inherent geometry of the helix. These coupled effects are more evident in the nonlocal solution, which captures the enhanced sensitivity of nanoscale structures to load transfer between bending and torsion.

**Figure 7:**
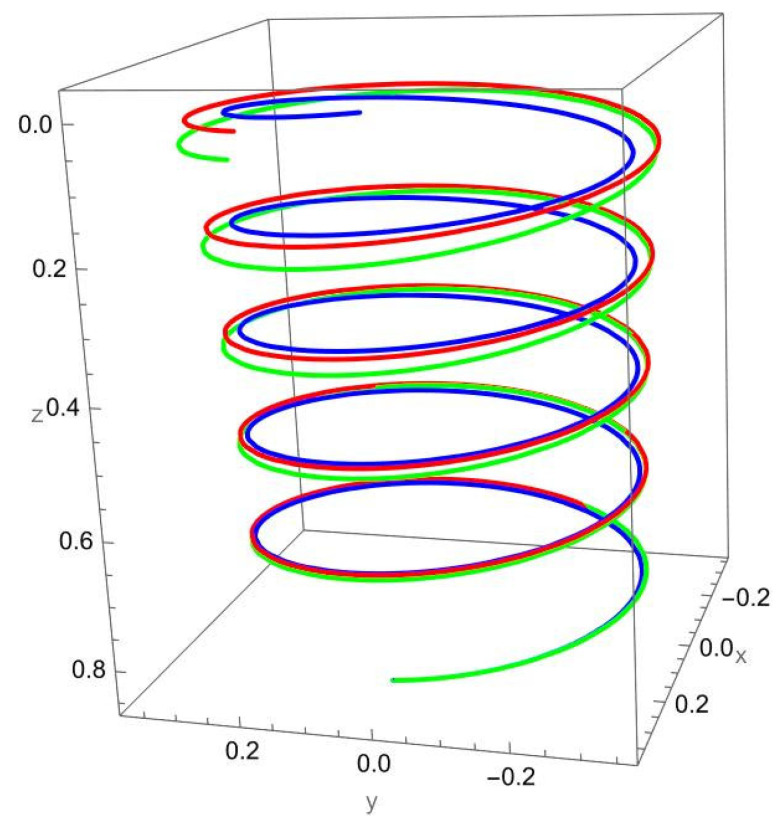
A cantilevered helical SWCNT beam under a concentrated binormal moment $M_{b}$ applied at the free end. Blue denotes the undeformed axis, red indicates the displacement predicted by local elasticity, and green shows the result of the nonlocal elasticity theory.

Figure 7 shows the deformation of the cantilevered helical SWCNT beam subjected to a binormal moment at the free end. This loading primarily induces bending about the binormal axis, but due to the helical geometry, the response involves coupled displacements and rotations in the normal and tangential directions as well.

The nonlocal prediction (green) exhibits significantly larger deflections and rotations than the local solution (red). This reflects the reduction in effective bending stiffness when nanoscale effects are included. At small values of R/γ the discrepancy between the two models is particularly pronounced, whereas at larger R/γ the two solutions converge, indicating reduced influence of nonlocality as the structural dimensions become much larger than the material length scale.

The geometry-driven coupling is evident in the deformation paths. The applied binormal moment not only produces curvature in the binormal plane but also amplifies twisting and secondary axial shifts, which are more strongly captured in the nonlocal solution. This behaviour underlines the enhanced role of curvature–torsion interactions at the nanoscale.

**Figure 8:**
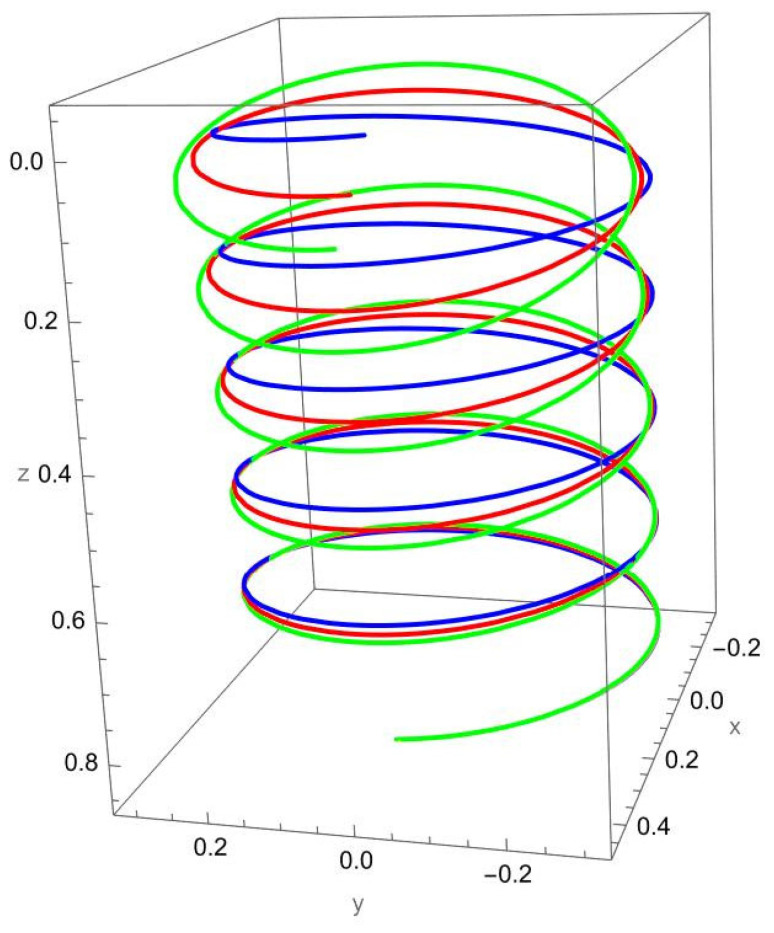
A cantilevered helical SWCNT beam under a concentrated tangential (torsional) moment $M_{t}$ applied at the free end. Blue denotes the undeformed axis, red indicates the displacement predicted by local elasticity, and green shows the result of the nonlocal elasticity theory.

Figure 8 illustrates the response of the cantilevered helical SWCNT beam subjected to a tangential (torsional) moment at the free end. This loading primarily excites twisting deformation along the helical axis, but due to the intrinsic coupling of curvature and torsion in the geometry, the resulting deformation also contains significant bending components.

The comparison between local and nonlocal predictions shows that the nonlocal formulation produces markedly larger twisting displacements and rotations, especially for small values of the nonlocal parameter R/γ. This reflects the reduction in torsional stiffness captured by nonlocal elasticity. As R/γ increases, the predictions of the two models converge, demonstrating the reduced influence of nanoscale effects when the characteristic length scale becomes negligible relative to the helix dimensions.

Secondary deformation patterns are also apparent. The torsional moment induces lateral shifts in both the normal and binormal directions, which are amplified in the nonlocal solution. These additional displacement components highlight the strong geometric coupling in helices, where torsion cannot occur independently of bending.

**Figure 9:**
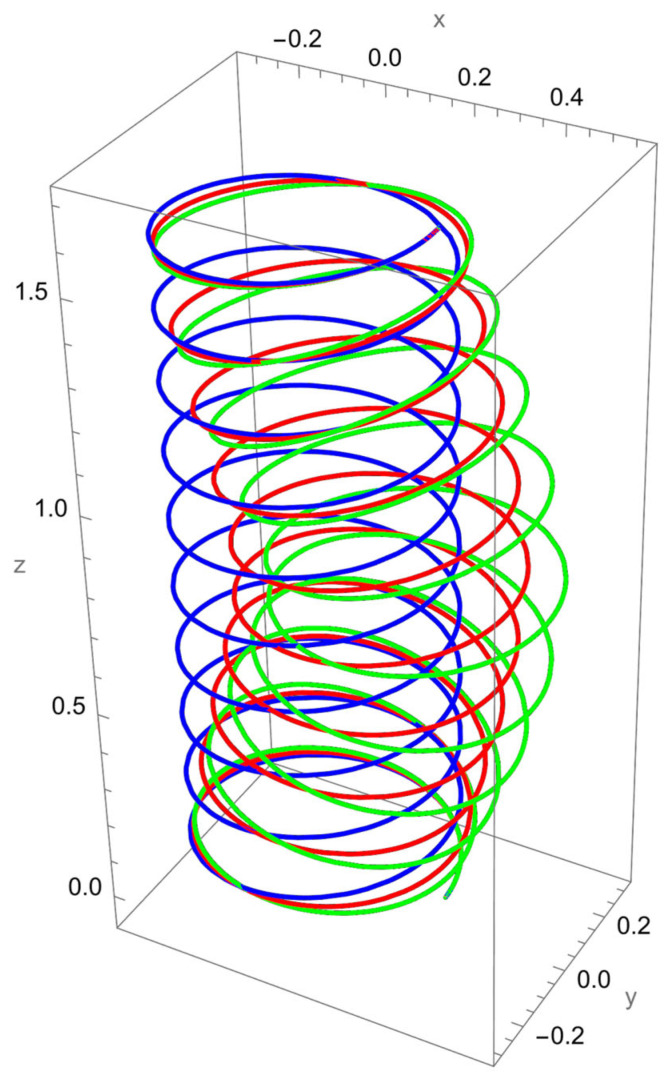
A clamped–clamped helical SWCNT beam (total arc length 20π ) subjected to a concentrated normal force $F_{n}$ applied at the midpoint (θ = 10π). Blue denotes the undeformed axis, red indicates the displacement predicted by local elasticity, and green shows the result of the nonlocal elasticity theory.

Figure 9 presents the deformation of a clamped–clamped helical SWCNT beam subjected to a concentrated normal force applied at its midpoint. Because of the boundary conditions, the deformation pattern is symmetric about the midspan, with deflections constrained at both ends. Both local and nonlocal elasticity theories predict the expected bending response in the normal direction, but the amplitude of displacement is noticeably higher for the nonlocal solution.

The difference between the two predictions reflects the reduced effective stiffness captured by nonlocal elasticity. At small values of R/γ, the nonlocal deformation is significantly larger, while at higher values of R/γ the results approach those of the local model. This behaviour confirms that scale-dependent effects dominate when the structural dimensions are comparable to the characteristic material length.

In addition to the dominant normal displacement, the helical geometry introduces tangential and binormal coupling effects. These are more pronounced in the nonlocal results, where the lateral deviations from the cylindrical axis are magnified. The amplification of coupled displacements highlights the sensitivity of helices to multiphysics interactions at the nanoscale.

**Figure 10:**
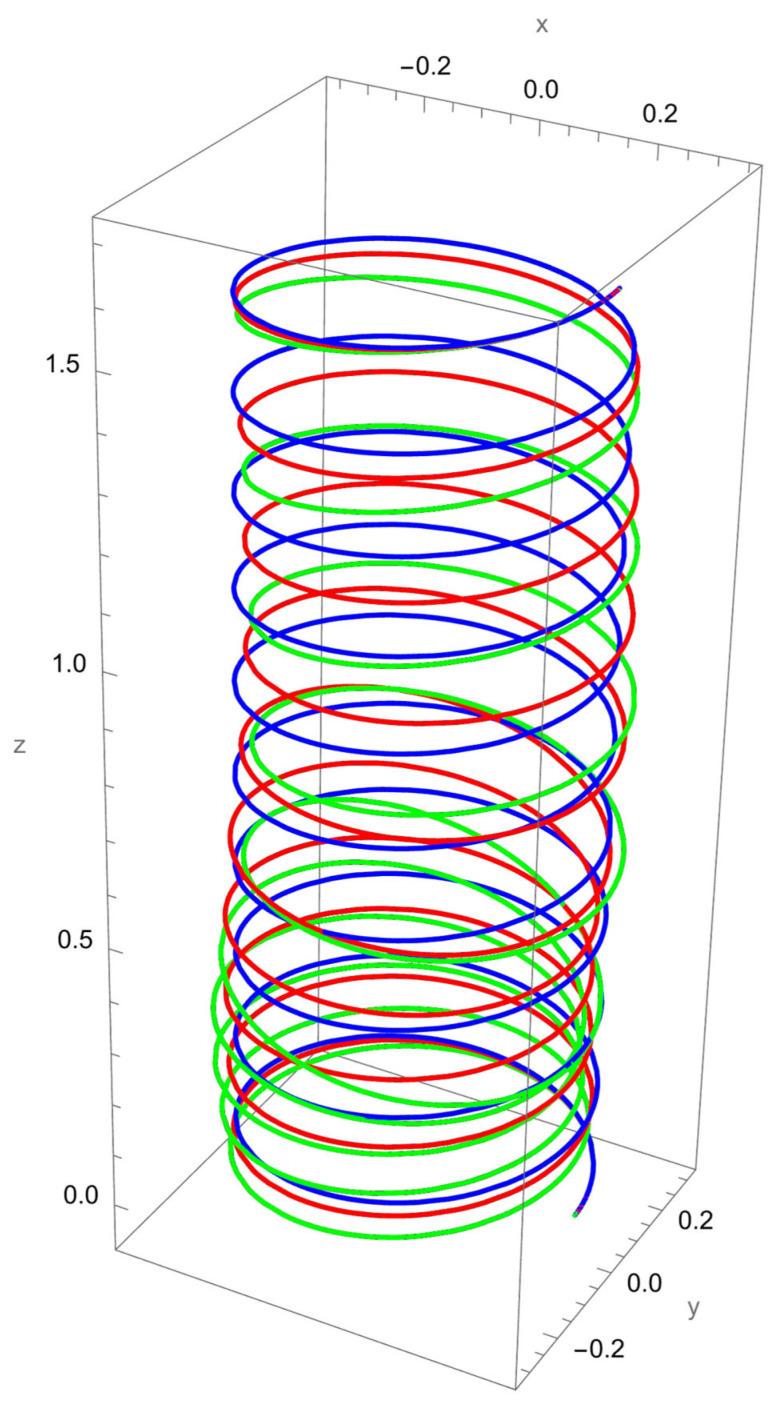
A clamped–clamped helical SWCNT beam (total arc length 20π) subjected to a concentrated binormal force $F_{b}$ applied at the midpoint (θ = 10π). Blue denotes the undeformed axis, red indicates the displacement predicted by local elasticity, and green shows the result of the nonlocal elasticity theory.

Figure 10 shows the deformation of a clamped–clamped helical SWCNT beam under a binormal force applied at the midpoint. Owing to the boundary constraints at both ends, the deformation is symmetric about the load application point, with lateral bending in the binormal direction forming the dominant response.

The comparison between local and nonlocal solutions reveals significant differences in the predicted amplitudes of displacement. The nonlocal solution (green) consistently produces larger lateral deflections than the local model (red), reflecting the reduction in effective bending stiffness at the nanoscale. This discrepancy is most pronounced for small values of the nonlocal parameter R/γ, while convergence between the two models occurs as R/γ increases.

In addition to the primary binormal deflection, the helical geometry introduces tangential and normal displacement components. These coupled effects are amplified in the nonlocal solution, where the twisting and lateral deviations become more pronounced. This again highlights that nanoscale interactions strengthen curvature–torsion coupling in helical structures.

**Figure 11:**
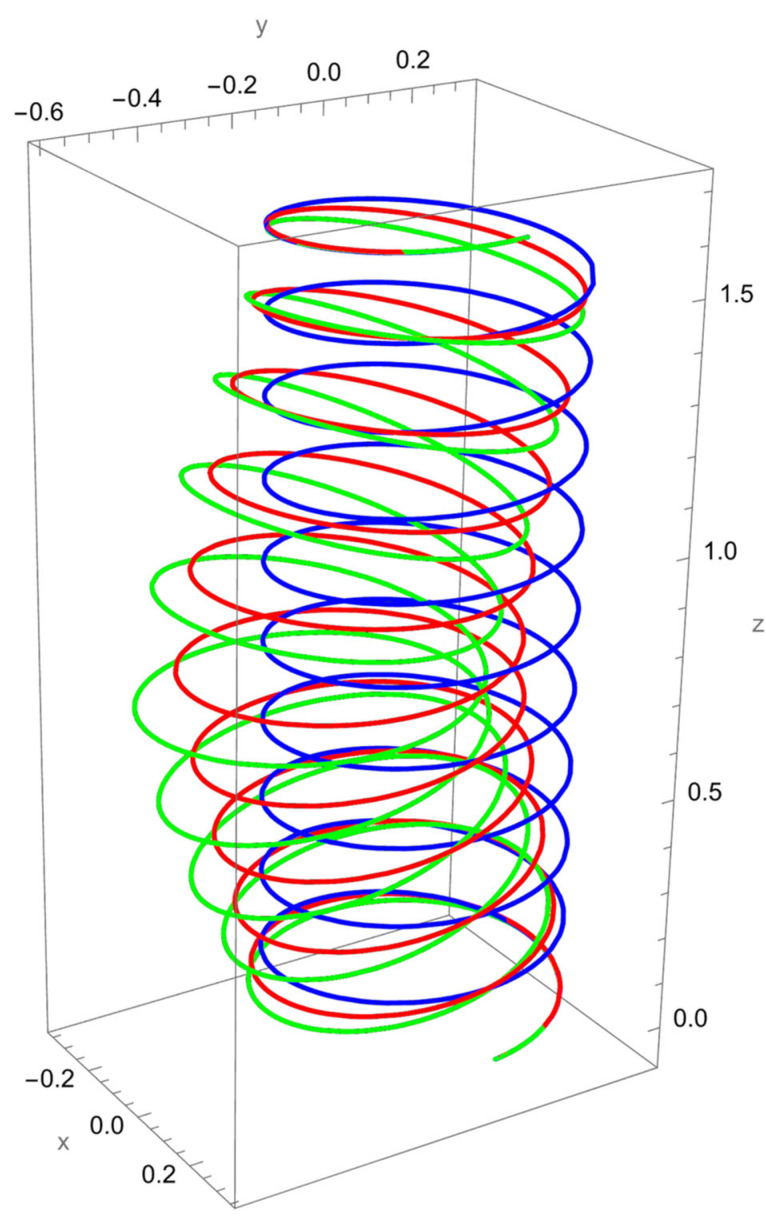
A clamped–clamped helical SWCNT beam (total arc length 20π) subjected to a concentrated tangential force $F_{t}$ applied at the midpoint (θ = 10π). Blue denotes the undeformed axis, red indicates the displacement predicted by local elasticity, and green shows the result of the nonlocal elasticity theory.

Figure 11 depicts the deformation of a clamped–clamped helical SWCNT beam subjected to a tangential force applied at its midpoint. The clamped supports enforce zero displacements and rotations at both ends, resulting in a symmetric deformation pattern about the load application point. The applied tangential force primarily excites torsional motion, but the helical geometry ensures strong coupling with bending displacements in both the normal and binormal directions.

The nonlocal model (green) predicts larger twisting and lateral deviations compared to the local model (red), reflecting the reduction in torsional stiffness due to nanoscale interactions. At small values of R/γ, this amplification is particularly evident, while for larger R/γ the two predictions approach one another, demonstrating the diminishing importance of nonlocality when the characteristic length scale is negligible compared to beam dimensions.

The coupling effects induced by tangential loading are amplified in the nonlocal solution. In addition to torsional deformation about the cylindrical axis, the structure experiences secondary lateral displacements, which are underestimated by the local model. This underlines the necessity of accounting for size-dependent effects when modelling nanoscale helices subjected to torsion-dominated loads.

**Figure 12:**
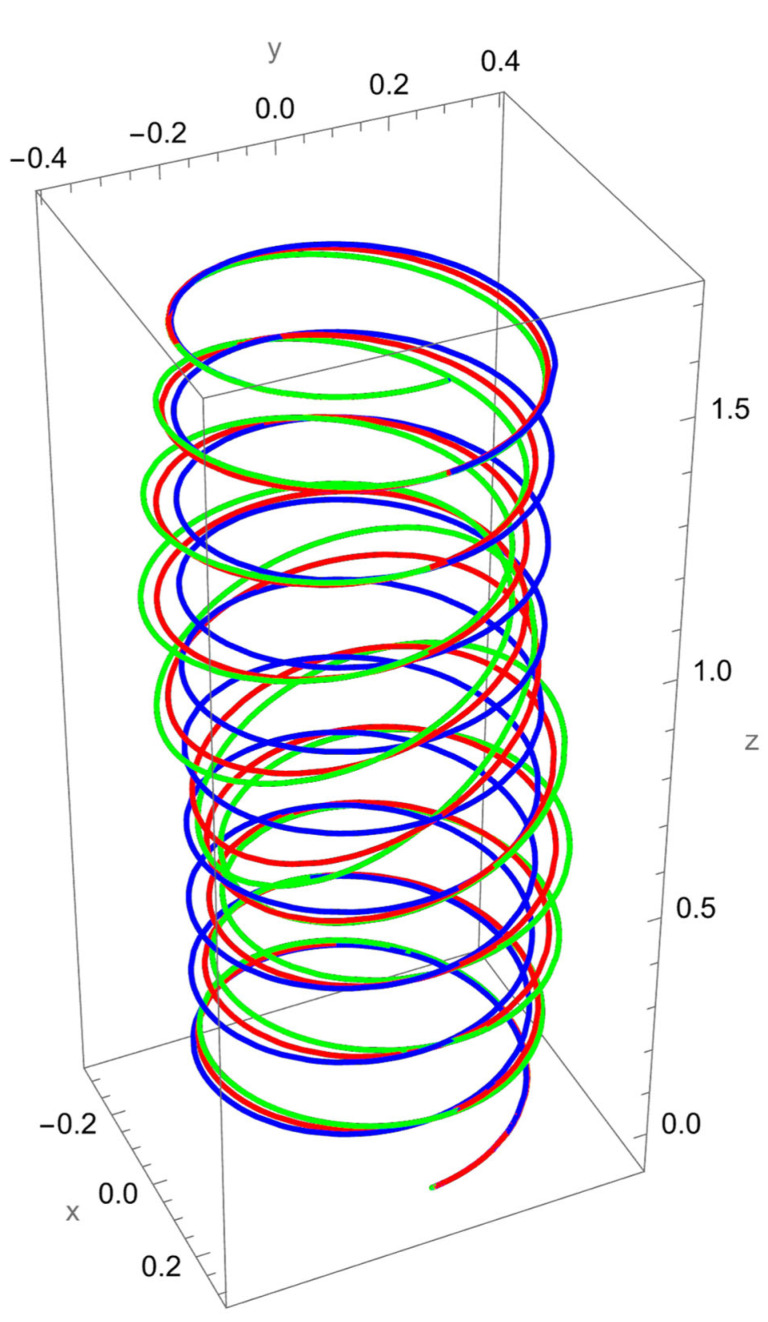
A clamped–clamped helical SWCNT beam (total arc length 20π) subjected to a concentrated normal moment $M_{n}$ applied at the midpoint (θ = 10π). Blue denotes the undeformed axis, red indicates the displacement predicted by local elasticity, and green shows the result of the nonlocal elasticity theory.

Figure 12 shows the deformation of a clamped–clamped helical SWCNT beam under a normal moment applied at its midpoint. The boundary conditions impose zero displacements and rotations at both ends, producing a symmetric deformation pattern centred on the load application point. The primary effect of the applied moment is bending about the normal axis, although the helical geometry ensures additional coupled displacements in the tangential and binormal directions.

The nonlocal solution (green) exhibits consistently larger rotations and displacements than the local model (red). This difference is most evident when the nonlocal parameter R/γ is small, reflecting the strong influence of size-dependent softening at the nanoscale. As R/γ increases, the two predictions converge, indicating that the role of nonlocal effects diminishes when the structural dimensions become large relative to the characteristic material length.

Coupling effects are also evident. In addition to the primary normal bending response, the helix undergoes secondary twisting and lateral shifts that are more pronounced in the nonlocal solution. These effects highlight the importance of including nonlocal elasticity in order to capture the full deformation complexity of nanoscale helical beams under moment-driven loading.

**Figure 13:**
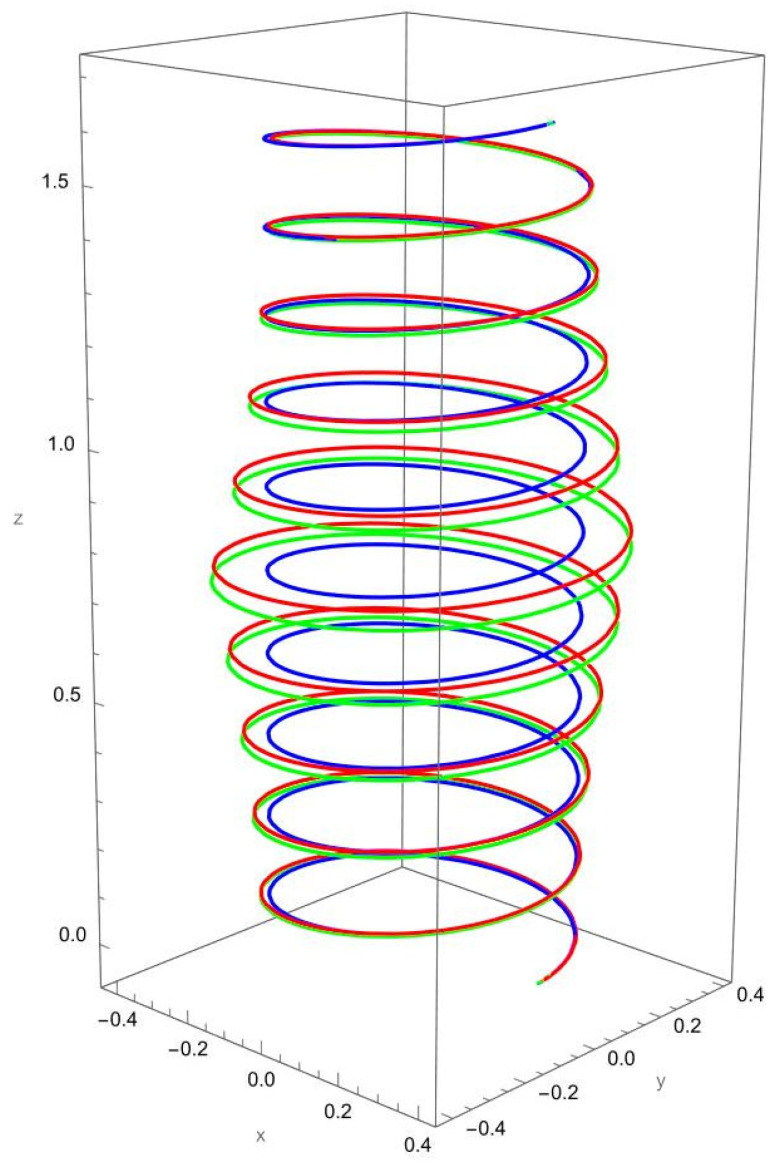
A clamped–clamped helical SWCNT beam (total arc length 20π) subjected to a concentrated binormal moment $M_{b}$ applied at the midpoint (θ = 10π). Blue denotes the undeformed axis, red indicates the displacement predicted by local elasticity, and green shows the result of the nonlocal elasticity theory.

Figure 13 presents the deformation of a clamped–clamped helical SWCNT beam under a binormal moment applied at the midpoint. With both ends fully constrained, the deformation pattern is symmetric about the load application point. The applied moment primarily induces bending about the binormal axis, though the curved geometry of the helix naturally introduces additional torsional and lateral coupling.

The comparison of results shows that the nonlocal solution (green) predicts larger deflections and rotations than the local model (red). This difference is especially pronounced at smaller values of the nonlocal parameter R/γ, where nanoscale effects strongly reduce the effective bending stiffness. As R/γ increases, the two predictions converge, consistent with the diminishing role of nonlocal interactions at larger structural scales.

Beyond the primary binormal bending response, secondary deformation effects are also observed. The nonlocal model captures amplified twisting and lateral deviations, which are largely underestimated by the local theory. This highlights how nanoscale effects enhance geometric coupling and increase the overall compliance of the helical structure.

**Figure 14:**
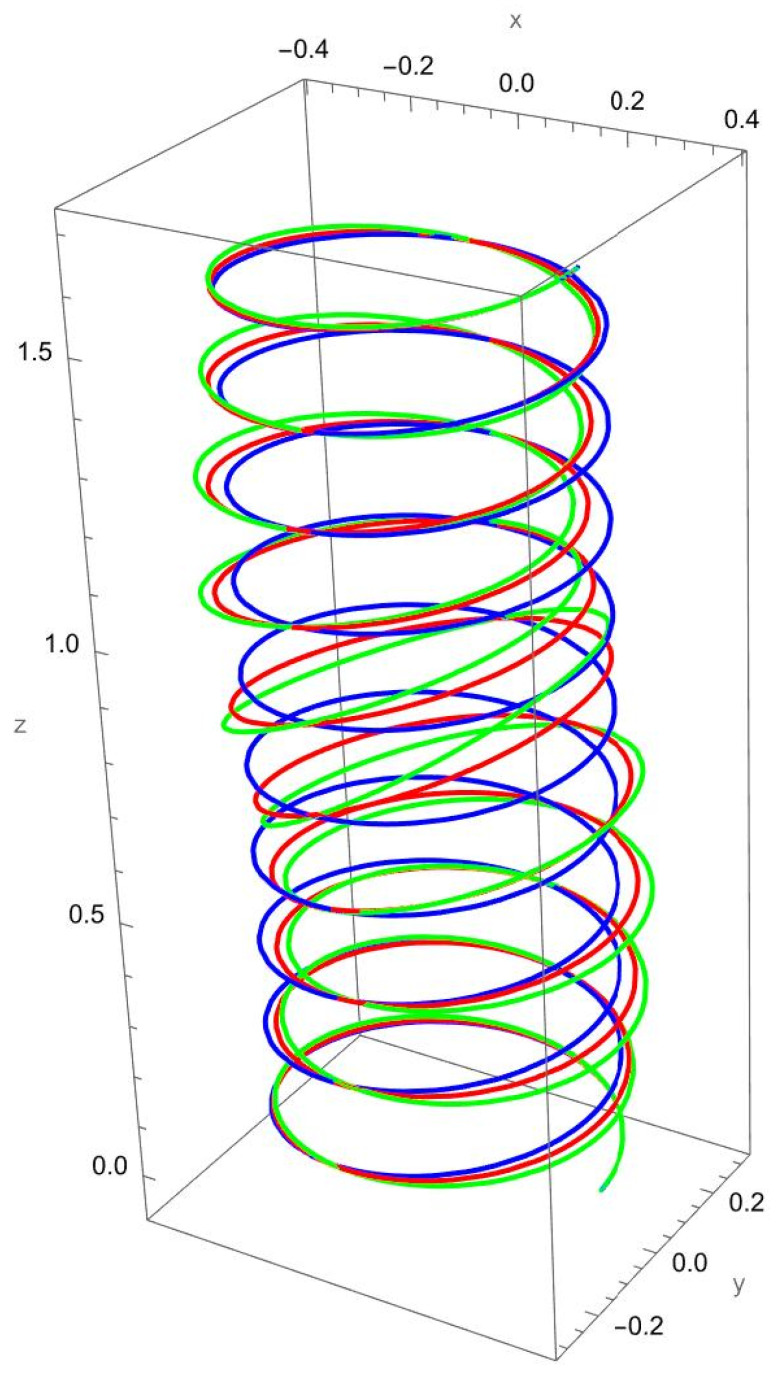
A clamped–clamped helical SWCNT beam (total arc length 20π) subjected to a concentrated tangential (torsional) moment $M_{t}$ applied at the midpoint (θ = 10π). Blue denotes the undeformed axis, red indicates the displacement predicted by local elasticity, and green shows the result of the nonlocal elasticity theory.

Figure 14 illustrates the deformation of a clamped–clamped helical SWCNT beam under a tangential (torsional) moment applied at its midpoint. The clamped supports at both ends enforce zero displacements and rotations, producing a symmetric twisting response about the midpoint. The primary effect of the applied moment is torsion about the helical axis, but the geometry couples this twisting with bending displacements in both the normal and binormal directions.

The comparison of local and nonlocal results again highlights the scale-dependent effects. The nonlocal solution (green) predicts considerably larger torsional rotations and lateral deviations compared to the local model (red). These differences are most pronounced when the nonlocal parameter R/γ is small, showing the reduced torsional stiffness associated with nanoscale interactions. At larger R/γ the two predictions converge, reflecting the reduced influence of nonlocality at larger scales.

Coupling effects are clearly visible: while the applied moment is tangential, the helix undergoes secondary bending displacements that are magnified in the nonlocal predictions. These additional deformation components emphasise the importance of incorporating nonlocal elasticity to fully capture the multiphysics interactions in nanoscale helical structures.

The deviations observed between local and nonlocal elasticity predictions are in line with earlier findings on curved nanobeams, where nonlocality was shown to reduce effective stiffness and amplify displacements [20,21,42,54,58,59]. However, the present results extend these insights by demonstrating that helical geometries not only soften under nonlocal effects but also exhibit strong torsion–bending coupling that is absent in simpler beam models. For example, the amplification of the binormal rotation $\phi_{b}$ under nonlocal assumptions highlights a cross-mode sensitivity that has not been emphasised in prior straight or mildly curved nanobeam analyses. This comparison indicates that nanoscale helices are more compliant and more strongly coupled in their deformation modes, which is a critical physical implication for the design of nanosprings, sensors, and flexible nano-actuators. While the present results establish a closed-form benchmark, full validation against molecular dynamics (MD) simulations of helical SWCNTs remains an important next step. Conducting such simulations under the exact boundary and loading conditions considered here is highly demanding in terms of computational cost and modelling setup, and is therefore beyond the scope of this analytical study. By expressing the solutions in nondimensional form, however, the framework is designed to facilitate direct comparison with existing and future MD datasets. This ensures that the present results can be readily used as a benchmark for multiscale validation in subsequent studies.

## 6. Conclusions

This study has presented exact analytical solutions for the static response of helical single-walled carbon nanotube (SWCNT) beams within the framework of Eringen’s nonlocal Euler–Bernoulli beam theory. By employing the Frenet frame to represent the helical geometry and applying the initial value method, closed-form relations for displacements and rotations were obtained under various concentrated loading conditions for both cantilevered and clamped–clamped boundary configurations.

The findings consistently demonstrate that nonlocal elasticity predicts larger displacements and rotations than the classical local theory, highlighting the softening of axial, flexural, and torsional stiffness that emerges when nanoscale effects are taken into account. The influence of the nonlocal parameter R/γ is strongest when the characteristic length is comparable to the dimensions of the structure, with local and nonlocal predictions converging as R/γ increases. Geometric parameters also play a decisive role in shaping the mechanical response: larger winding angles are associated with enhanced stiffness, while variations in pitch angle alter the balance between axial elongation and shortening. Tangential and torsional loadings revealed the greatest differences between local and nonlocal elasticity, underlining the strong coupling between torsion and bending inherent to helical geometries.

The closed-form analytical solutions obtained here provide benchmark reference data for the mechanics of nanoscale helical beams. They confirm the necessity of incorporating nonlocal elasticity into nanoscale modelling and offer a rigorous framework that can be used for validation of multiscale simulations, design optimisation, and experimental calibration in nanotechnology applications.

Looking ahead, this formulation can be extended to address dynamic behaviour, including vibrations, wave propagation, and transient responses, as well as the influence of temperature-dependent material properties and viscoelastic effects. Incorporating electromechanical, piezoelectric, or magneto-mechanical coupling would further enable the design of multifunctional nanosprings, sensors, and actuators. The approach may also be generalised to more complex architectures such as multi-walled CNTs, helical bundles, or CNT-reinforced composites, where inter-wall or inter-phase interactions become significant. Finally, correlation with molecular dynamics simulations and experimental studies will be essential to validate the predictions and calibrate the nonlocal length-scale parameters.

It should be noted that the present formulation is based on the Euler–Bernoulli beam assumption, which is most suitable for slender SWCNTs with high aspect ratios. For helices with larger pitch angles or lower aspect ratios, shear deformations and rotary inertia effects may become more significant. In such cases, a Timoshenko-type nonlocal helical beam model would provide a more accurate representation of the mechanics. Developing this extended formulation represents a natural direction for future work, in line with existing nonlocal Timoshenko beam studies for curved nanostructures.

Also, the present study is formulated within the framework of linear elasticity. Order-of-magnitude estimates of the Green–Lagrange strains under the largest load cases indicate values well within the limits of linear elasticity demonstrating the validity of this assumption. Nonetheless, for loading scenarios that produce larger strains, geometric nonlinearity should be considered in future work.

Beyond these modelling assumptions, several additional limitations should be highlighted. The current formulation does not account for van der Waals interactions between adjacent coils, which may influence the response in tightly wound helices. Thermal effects are neglected, even though they can significantly alter the effective stiffness of CNTs at the nanoscale. Finally, the study focuses exclusively on single-walled CNTs, and extensions to multi-walled structures remain an open area for further investigation.

In conclusion, this work establishes the first exact closed-form static analysis of nonlocal helical nanobeams. It provides a solid foundation for future investigations into their dynamic, thermal, and multiphysics responses and emphasises the importance of size-dependent mechanics for the accurate modelling and reliable design of nanoscale devices based on helical SWCNTs.

## Figures and Tables

**Table 1:** Normalised displacement and rotation responses of cantilevered helical SWCNT beams subjected to end-applied axial loads: comparison of local and nonlocal elasticity predictions for varying nonlocal parameter R/γ, winding angle θ, and pitch angle α.

θ	α (°)	$\frac{R}{\gamma }$	${\bar{u}}_{n}$	${\bar{u}}_{b}$	${\bar{u}}_{t}$	$\Omega_{b}$	$\Omega_{t}$
4π	5	1	−0.447169	−0.385396	−0.189402	0.185118	−0.364152
		5	−0.277218	0.018847	0.028176	0.049663	−0.005685
		10	−0.271907	0.031479	0.034975	0.045430	0.005517
		Local	−0.270136	0.035690	0.037241	0.044019	0.009251
	10	1	−0.717249	−0.143186	−0.714675	−0.412358	−0.017279
		5	−0.083025	−0.022902	−0.053474	−0.026794	0.011575
		10	−0.063205	−0.019143	−0.032811	−0.014745	0.012477
		Local	−0.056599	−0.017890	−0.025924	−0.010729	0.012778
	15	1	1.141435	−0.002010	0.636183	0.464196	−0.029617
		5	0.116157	−0.011459	0.054075	0.031576	0.010393
		10	0.084117	−0.011754	0.035884	0.018057	0.011643
		Local	0.073437	−0.011852	0.029821	0.013550	0.012060
10π	5	1	−0.822377	−0.410876	−0.153064	0.334183	−0.648534
		5	−0.509823	0.024733	0.042914	0.098631	−0.035122
		10	−0.500056	0.038346	0.049038	0.091270	−0.015953
		Local	−0.496800	0.042883	0.051080	0.088816	−0.009563
	10	1	−0.424552	−0.083568	−0.745981	−0.402440	−0.112901
		5	−0.049144	−0.010252	−0.063326	−0.034860	−0.005467
		10	−0.037412	−0.007960	−0.041993	−0.023373	−0.002100
		Local	−0.033502	−0.007197	−0.034882	−0.019544	−0.000990
	15	1	0.965367	0.073051	0.867905	0.556787	−0.165598
		5	0.098240	0.001411	0.074478	0.046980	−0.008565
		10	0.071142	−0.000828	0.049684	0.031049	−0.003658
		Local	0.062110	−0.001574	0.041419	0.025739	−0.002022

## Data Availability

No new data were created or analyzed in this study. Data sharing is not applicable to this article.
